# Omentin-1 Modulates Macrophage Function *via* Integrin Receptors αvβ3 and αvβ5 and Reverses Plaque Vulnerability in Animal Models of Atherosclerosis

**DOI:** 10.3389/fcvm.2021.757926

**Published:** 2021-11-02

**Authors:** Xuze Lin, Yan Sun, Shiwei Yang, Mengyue Yu, Liu Pan, Jie Yang, Jiaqi Yang, Qiaoyu Shao, Jinxing Liu, Yan Liu, Yujie Zhou, Zhijian Wang

**Affiliations:** ^1^Department of Cardiology, Beijing Anzhen Hospital, Capital Medical University, Beijing, China; ^2^State Key Laboratory of Cardiovascular Disease, Department of Cardiology, National Center for Cardiovascular Diseases, Fuwai Hospital, Chinese Academy of Medical Science and Peking Union Medical College, Beijing, China; ^3^Peking Union Medical College, Beijing, China; ^4^Chinese Academy of Medical Science, Beijing, China; ^5^Beijing Institute of Heart Lung and Blood Vessel Disease, Beijing, China

**Keywords:** plaque vulnerability, adipokine, integrin, atherosclerosis, macrophage

## Abstract

**Backgrounds:** Omentin-1 is a novel cytokine that is primarily released by the epicardial adipose tissue. Molecular structure analysis revealed that it contained a fibrinogen-like domain. Clinical studies have demonstrated that the expression of omentin-1 is tightly associated with the development of cardiovascular diseases, but the receptor by which omentin-1 modulates macrophage function has not been identified yet.

**Objective:** This study sought to investigate the effect of omentin-1 on already-established atherosclerosis (AS) lesions in both ApoE−/− and Ldlr^−/−^ mice and further, study its underlying mechanisms.

**Methods and Results:** We investigated the effect of omentin-1 on the plaque phenotype by implanting a minipump in ApoE−/− and Ldlr^−/−^ mice. *In vivo* studies showed that the infusion of omentin-1 increased the collagen content and mitigated the formation of the necrotic core in both animal models. Immunohistochemistry and immunofluorescence analysis revealed that omentin-1 suppressed inflammatory cytokines expression, macrophage infiltration, and apoptosis within the plaque. An immunoprecipitation experiment and confocal microscopy analysis confirmed the binding of omentin-1 to the integrin receptors αvβ3 and αvβ5. The cell studies demonstrated that omentin-1 suppressed the apoptosis and inflammatory cytokines expression induced by the oxidized low-density lipoprotein in the macrophage. In addition, omentin-1 promoted the phosphorylation of the integrin-relevant signaling pathway as well as the Akt and AMPK in the macrophage. The addition of the inhibitor of the integrin receptor or interfering with the expression of the integrin subunit αv (ITGAV) both significantly abrogated the bioeffects induced by omentin-1. A flow cytometry analysis indicated that the antibodies against αvβ3 and αvβ5 had a competitive effect on the omentin-1 binding to the cell membrane.

**Conclusions:** The administration of adipokine omentin-1 can inhibit the necrotic cores formation and pro-inflammatory cytokines expression within the AS lesion. The mechanisms may include the suppression of apoptosis and pro-inflammatory cytokines expression in the macrophage by binding to the integrin receptors αvβ3 and αvβ5.

## Introduction

Coronary artery atherosclerosis (AS), which is characterized by the formation of an atherosclerotic lesion in the lumen epicardial arteries, frequently induces the stenosis of nourishing vessels of the myocardium and eventually gives rise to the myocardial ischemia (1). Myocardial infarction is majorly caused by acute coronary syndrome (ACS), and plaque vulnerability is considered to play an important role in the induction of ACS. Many studies have demonstrated that macrophage infiltration, coupled with its apoptosis and inflammatory cytokine secretion, contributes to plaque instability and finally leads to plaque rupture (2, 3). Therefore, the macrophage is now believed to be a potential therapeutic target in treating advanced AS plaque (4).

Several *in vivo* and *in vitro* studies revealed that the adipose tissue abundantly secretes bioactive molecules, which are termed adipokines, and impacts the metabolism profile of adjacent and remote organs by the paracrine and endocrine pathways (5). Omentin-1, which is an adipokine mainly expressed by the visceral and epicardial adipose tissue (EAT), is composed of 313 amino acids and is considered to be a hydrophilic protein. Gaborit et al. reported that the expression level of omentin-1 in the EAT is 12-fold higher than that in subcutaneous fats, and, surprisingly, our previous research indicated that the omentin-1 expression is much lower in the EAT adjacent to the coronary stenotic segments (6, 7).

Previous biochemical studies have demonstrated that omentin-1 can produce favorable effects on the cardiovascular system by promoting vasodilation in isolated vessels and inhibiting the growth and migration of vascular smooth muscle cells (VSMCs) (8, 9). The study by Mizuho et al. provided evidence that omentin-1 generated beneficial effects on macrophages mainly by activating the phosphatidylinositol 3-kinase (PI3K)/protein kinase B (Akt) signaling pathway (10). Besides, omentin-1 also suppressed the expression of the intercellular cell adhesion molecule-1 (ICAM-1) and vascular cell adhesion molecule-1 (VCAM-1) in the human umbilical vein endothelial cells (HUVECs), which contributed to the reduction in the adhesion of monocytes to HUVECs (11). However, the cellular receptor by which omentin-1 exerts its conducive function has not been elucidated yet.

Integrin family receptors, which are a cluster of transmembrane receptors consisting of 18 α and 8 β subunits, serve an important role in the cellular crosstalk with its microenvironment. They frequently recognize and bind to extracellular matrix (ECM) components, including fibronectin, vitronectin, osteopontin, and fibrinogen. Integrin αvβ3 and αvβ5, which are heterodimers composed of αvβ3 and β5 subunits, have been reported to be associated with the development of AS (12, 13). Though the receptor of omentin-1 has not been fully identified yet, we noticed that omentin-1 has been reported to have a fibrinogen-like domain, which might be the integrin-binding motif on it (14). Encouraged by these profound discoveries, we decided to explore whether adipokine omentin-1 can directly bind to the integrin αvβ3 or αvβ5 in macrophage-derived foam cells and whether it can affect the phenotype of established plaques *in vivo* by regulating foam cell functions.

In this research, we used apolipoprotein E-deficient (ApoE^−/−^) mice and low-density lipoprotein receptor-deficient (Ldlr^−/−^) mice with an ALZET minipump implantation to investigate the therapeutic effect of omentin-1 on the already-established AS plaques. In cell studies, we applied RAW264.7 and THP1 cell lines to construct the macrophage cell model *in vitro*. Oil red O staining and immunofluorescent technology were used to confirm the lipid ingestion and phagocytosis activity of cells. To assess the role played by integrin αvβ3 and αvβ5 in transducing the signal of omentin-1, we used cilengitide to inhibit the integrin receptors and applied gene interfering technology *in vitro* to knock down the integrin subunit αv (ITGAV) expression.

## Methods

A detailed description of the methods is included in the Supplementary Material and Research resources.

### Animal Model

The animal experiments were performed in accordance with the Institutional Animal Care and Use of Laboratory Animals and were approved by the Capital Medical University Animal Care and Use Committee. Additionally, all of the animal experiments performed conformed with the European community guiding principles in the care and use of animals (2010/63/UE). A total of 58 male ApoE−/− mice (C57BL/6 background) at the age of 8 weeks were purchased from Beijing Vital River Laboratory Animal Technology and a total of 40 male Ldlr−/− mice (C57BL/6 background) at the age of 10 weeks were purchased from Guangzhou Cyagen Biosciences Inc. The ApoE−/− mice were fed on a western diet for 12 weeks and the Ldlr−/− mice were fed on a western diet for 8 weeks to build up the AS lesion. The groups without minipump implantation were euthanized under deep anesthesia [induced by intraperitoneal injection with an overdose of pentobarbital sodium (200 mg/kg)] to get their aortas and blood samples. The rest of the mice were randomly divided into three groups: (i) controls (infused with normal saline), (ii) low dose omentin-1-treated group (infused with omentin-1 at the rate of 1 μg/kg/h *via* ALZET minipump), (iii) high dose omentin-1-treated group (infused with omentin-1 in the rate of 5 μg/kg/h *via* the ALZET minipump). ALZET minipumps (ALZET Model 2004; Cupertino, California, United States) were used to achieve the continuous infusion of the flag-tagged omentin-1 (Abcam, ab157030, UK) solution into the mouse jugular vein. For implanting the minipumps, anesthesia was induced to the mice by inhaling 2% isoflurane and maintained by inhaling 1.5% isoflurane. Once anesthetized, the mice were instrumented with a polyurethane catheter (ALZET, Cat No: 0007700; Cupertino, California, United States) implanted in the right jugular vein. The catheter was attached to an ALZET minipump, which was implanted subcutaneously. All of the remaining mice were fed on a western diet after the implantation of the minipumps. After 3 weeks of infusion, their blood pressure and body weight were measured before receiving euthanasia under deep anesthesia (the method was described as above). The blood and whole aorta sample of every mouse were obtained. The fasting plasma glucose, triglyceride, total cholesterol, low-density lipoprotein (LDL) cholesterol, and high-density (HDL) cholesterol were measured using enzymatic methods. The omentin-1 concentration of the plasma was measured by enzyme-linked immunoassay (ELISA) analysis (Biovendor, RD191100200R, Czech Republic).

### Mouse Aortic Root Atherosclerotic Lesion Assessment

Eight micrometers (8 mm) cryosections were taken from the entire region of the valve leaflet and every 10th section (80 μm) was subjected to Oil red O staining and hematoxylin counterstaining. Images were captured under an identical microscope. All of the sections were coded and analyzed blind. Three sections from different levels of the aortic root in each mouse were obtained and subjected to hematoxylin and eosin (HE) staining (Solarbio Life Science, G1262, Beijing, China) and Masson's trichrome staining (Solarbio Life Science, G1340, Beijing, China).

The macrophage content of the plaque was expressed as the proportion of CD68+ cells. To determine the expression of the pro-inflammatory cytokines within the atherosclerotic lesion, immunohistochemistry analysis, and western blot were performed by using antibodies against TNF-α (Abcam, ab9739, UK), and IL-1β (Abcam, ab9722, UK). The mouse IgG isotype (R&D, MAB002, USA) was used as a negative control. To detect the colocalization of the exogenous omentin-1 to the plaque integrin αvβ3 and αvβ5, immunofluorescence co-localization analysis was performed by applying antibodies against flag tag (Abcam, ab205606, UK), integrin αvβ3 (Santa Cruz, sc-7312), integrin αvβ5 (Santa Cruz, sc-13588) and F4/80 (Abcam, ab16911, UK).

The cell apoptosis in the plaque was detected by using an *in situ* cell death detection kit (Roche, 11684795910, Switzerland), and the cells undergoing apoptosis were quantified by counting the TUNEL+ cells.

About 6 μm of aortic root tissue was taken from the subjects for western blot analysis. The adherent tissue of the vessel was removed carefully before adding a lysis buffer. The protein concentration of each sample was quantified and equilibrated before undergoing western blotting.

### Cell Culture and Functional Assays

The RAW264.7 and THP1 cell lines were purchased from Cyagen Bioscience (Guangzhou, China). The culture condition was described in the Supplementary Materials. Functional assays including apoptosis, foam cell formation, and small interfering RNA (siRNA) transfection were performed as described in the Supplementary Materials. The nucleotide sequence of siRNAs and primers is shown in Tables 1, 2. To investigate the interaction between omentin-1 and the integrin receptors αvβ3 and αvβ5, we performed co-immunoprecipitation (IP) and confocal microscopy analysis as described in the Supplementary Material. To examine whether the neutralizing antibody against αvβ3 or αvβ5 had a competitive effect on the binding of omentin-1 to integrin receptors, we performed a flow cytometry assay, which is described in the Supplementary Materials.

**Table 1:** List of nucleotide sequence of small interfering RNA.

**Names**	**Forward sequence**	**Reverse sequence**
NC (negative control)	UUCUCCGAACGUGUCACGUTT	ACGUGACACGUUCGGAGAATT
ITGAV siRNA	GACCCGUUGUCACUGUAAATT	UUUACAGUGACAACGGGUCTT

**Table 2:** List of primer sequence used for RT-qPCR.

**Gene**	**Forward sequence (5^**′**^ to 3^**′**^)**	**Reverse sequence (5^**′**^ to 3^**′**^)**
ITGAV	GTGTGAGGAACTGGTCGCCTAT	CCGTTCTCTGGTCCAACCGATA
GAPDH	CATCACTGCCACCCAGAAGACTG	ATGCCAGTGAGCTTCCCGTTCAG

### Statistical Analysis

The results were presented as mean ± SEM for the continuous data and as frequencies for the categorical data. An unpaired two-tailed Student *t*-test was used for comparison between two groups. A one-way ANOVA followed by a Bonferroni multiple comparisons post-test was performed to assess the significance of deviation when three or more groups were involved. The χ^2^ test was used to compare the categorical data. All data were analyzed by GraphPad Prism 7 (GraphPad Software Inc., San Diego, California, United States) and SPSS 26 (IBM, Armonk, New York, United States). *P* < 0.05 were considered as statistically significant. The *P*-values are given in the figures. The representative images were selected as those that show values close to the means of the results obtained from all the analyzed samples.

## Results

### Assessment of Omentin-1 Retention in Atherosclerotic Lesion

After being treated by flag-tagged omentin-1 for another 3 weeks, the omentin-1 concentration in the plasma of the mice was measured by ELISA analysis, and the retention of omentin-1 in their aortic root lesion was examined by immunofluorescence analysis. The result of the ELISA showed that the intravenous infusion of exogenous omentin-1 significantly elevated the plasma omentin-1 level in the ApoE−/− mice and Ldlr−/− mice (Tables 3, 4). The ApoE−/− and Ldlr−/− mice treated by a high dose omentin-1 (5 μg/kg/h) exhibited elevated omentin-1 retention in their plaque area when compared with the counterparts treated with low dose omentin-1 (1 μg/kg/h) or normal saline (NS) (Supplementary Figures 1A,B, 2A,B). Moreover, the biomedical analysis of the blood samples indicated that the serum level of omentin-1 was positively associated with the rate of omentin-1 infusion, while the other risk factors of AS remained unaffected (Tables 3, 4). These results illustrated that the intravenous infusion of omentin-1 solution can successfully bring omentin-1 to the blood and AS plaque of animals.

**Table 3:** Characteristics of ApoE^−/−^ mouse.

	**Before surgery**	**NS infusion**	**Omentin-1** **(1 μg/kg/h)**	**Omentin-1** **(5 μg/kg/h)**
Male (%)	100	100	100	100
Age (weeks)	20	23	23	23
Body weight (g)	29.1 ± 0.7	30 ± 0.64	29.4 ± 0.65	30.4 ± 0.8
Systolic BP (mmHg)	102.4 ± 1.4	103.7 ± 1.3	103 ± 1.7	104.1 ± 1.5
Diastolic BP (mmHg)	78.3 ± 1.8	81 ± 1.9	78.1 ± 2.1	79.2 ± 2.2
Glucose (mg/dl)	192.1 ± 8.7	207.1 ± 5.2	199.7 ± 4.8	196.2 ± 5.2
Triglyceride (mg/dl)	66.7 ± 2.1	65.4 ± 2.0	64.3 ± 1.8	64.0 ± 2.0
Total cholesterol (mg/dl)	1713 ± 41.2	1723 ± 38.0	1655 ± 32.3	1594 ± 44.7
LDL-C (mg/dl)	274 ± 8.5	251.7 ± 8.5	248.4 ± 9.0	238.7 ± 9.9
HDL-C (mg/dl)	36.9 ± 0.7	36.5 ± 0.6	36.6 ± 0.6	36.4 ± 0.7
Human omentin-1 (ng/ml)	1.19 ± 0.03	1.2 ± 0.05	59.82 ± 4.3^*,†^	433 ± 19.1^**,‡,¶^
*n*	10	16	16	16

*Baseline characteristics of ApoE^−/−^ mouse was recorded. And the blood samples of them were collected and subjected to biochemical analysis. Before surgery represents the group without ALZET minipump implantation. NS infusion represents the group infused with normal sodium solution (vehicle). Omentin-1 represents the group infused with omentin-1-containing solution [*

* *P < 0.05*,

** *P < 0.0001 vs. the group without ALZET minipump implantation (Before surgery)*;

† *P < 0.05*,

‡ *P < 0.0001 vs. the group infused with normal sodium solution (NS infusion)*;

¶ *P < 0.0001 vs. the group infused with low concentration of omentin-1 solution (omentin-1 1 μg/kg/h)]*.

**Table 4:** Characteristics of Ldlr^−/−^ mouse.

	**Before Surgery**	**NS infusion**	**Omentin-1** **(1 μg/kg/h)**	**Omentin-1** **(5 μg/kg/h)**
Male (%)	100	100	100	100
Age (weeks)	18	21	21	21
Body weight (g)	30.6 ± 0.8	30.6 ± 0.7	30.3 ± 0.7	29.4 ± 0.6
Systolic BP (mmHg)	104.9 ± 2.1	103 ± 2.0	106.2 ± 2.5	105.9 ± 3.0
Diastolic BP (mmHg)	77.9 ± 1.1	79.6 ± 1.3	79.3 ± 1.3	77.7 ± 1.3
Glucose (mmol/L)	227.9 ± 7.0	219.7 ± 6.7	216.9 ± 6.2	216.7 ± 6.3
Triglyceride (mmol/L)	325.4 ± 11.6	338.7 ± 12.0	323.0 ± 10.7	322.0 ± 11.2
Total cholesterol (mmol/L)	1772 ± 46.0	1756 ± 54.6	1714 ± 49.6	1687 ± 52.9
LDL-C(mmol/L)	396.5 ± 17.4	412.1 ± 18.5	432 ± 19.2	427.8 ± 23.9
HDL-C(mmol/L)	125.2 ± 3.0	126 ± 3.0	122.9 ± 4.0	122.4 ± 2.6
Human omentin-1 (ng/ml)	2.19 ± 0.22	2.38 ± 0.28	43.17 ± 2.88^*,†^	385 ± 15.90^**,‡,¶^
*n*	10	10	10	10

*Baseline characteristics of Ldlr^−/−^ mouse was recorded. And the blood samples of them were collected and subjected to biochemical analysis. Before surgery represents the group without ALZET minipump implantation. NS infusion represents the group infused with normal sodium solution (vehicle). Omentin-1 represents the group infused with omentin-1-containing solution [*

* *P < 0.05*,

** *P < 0.0001 vs. the group without ALZET minipump implantation (Before surgery)*;

† *P < 0.05*,

‡ *P < 0.0001 vs. the group infused with normal sodium solution (NS infusion)*;

¶ *P < 0.0001 vs. the group infused with low concentration of omentin-1 solution (omentin-1 1 μg/kg/h)]*.

### Omentin-1 Infusion Modulates Plaque Phenotype in ApoE−/− and Ldlr−/− Mice

**Figure 1:**
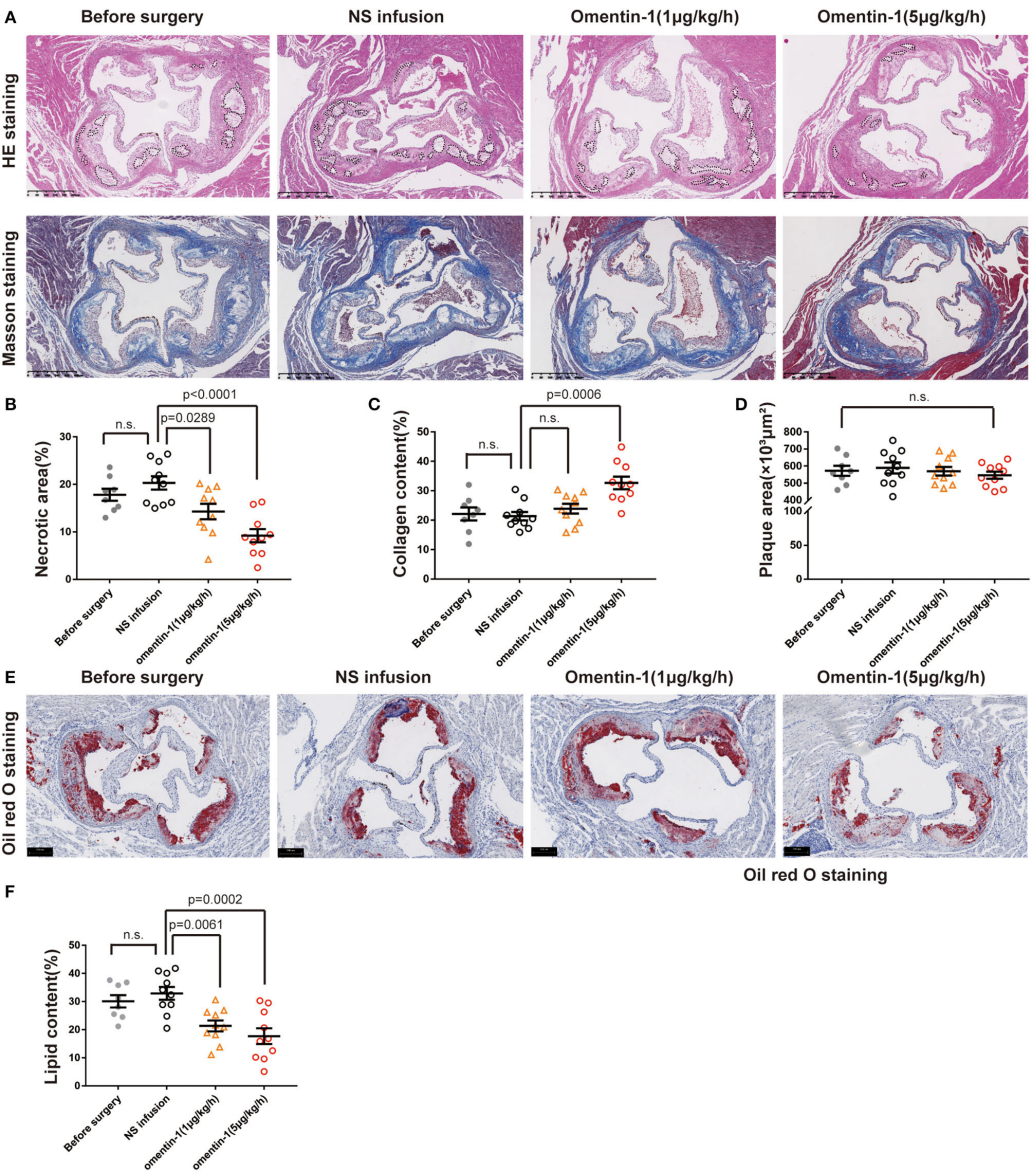
Infusion of omentin-1 enhanced plaque stability in ApoE^−/−^ mice. **(A)** Representative images of aortic root lesion sections of ApoE^−/−^ mice stained by hematoxylin and Masson's trichrome. **(B)** Graph shows the formation of necrotic cores. The formation of necrotic cores (pointed out by dot line) was assessed by calculating the proportion of necrosis area in the plaque (n_1_ = 8, n_2,3,4_ = 10). **(C)** Graph shows the collagen content of atherosclerosis (AS) plaque. Collagen content was expressed in the proportion of collagen fiber (blue) in the plaque area (n_1_ = 8, n_2,3,4_ = 10). **(D)** Graph shows the size of the AS lesion in each group. No significant difference was found between the experimental groups and control groups (n_1_ = 8, n_2,3,4_ = 10). **(E)** Representative images of the aortic root sections of mice stained by oil red O. **(F)** Graph show the lipid content (red) in the plaque area. The lipid content was expressed in the proportion of oil red positive area in plaque area (n_1_ = 8, n_2,3,4_ = 10). All data in this figure were presented as mean ± SEM [n.s., non-significant; n_1_, the number of subjects in Before Surgery group; n_2_, the number of subjects in normal saline (NS) infusion group; n_3_, the number of subjects in group infused with omentin-1 (1 μg/kg/h); n_4_, the number of subjects in group infused with omentin-1 (5 μg/kg/h)].

After being given by NS or omentin-1 solution for 3 weeks, the aortic root lesion of the ApoE−/− and Ldlr−/− mice were stained with an HE stain reagent and Masson's trichrome stain reagent (Figure 1A and Supplementary Figure 4A). The HE staining and Masson's trichrome staining of the aortic root section from the ApoE−/− and Ldlr−/− mice indicated that a high dose of omentin-1 solution (5 μg/kg/h) treatment significantly suppressed the necrotic core formation and elevated the collagen content in the AS lesion when compared with the counterpart that was treated with NS (Figures 1B,C and Supplementary Figures 4B,C). The low dose of omentin-1 treatment (1 μg/kg/h) helped to ameliorate the necrotic formation in the ApoE−/− mice but not in the Ldlr−/− mice (Figures 1B,C and Supplementary Figures 4B,C). However, the 3-week treatment of omentin-1 did not reverse the AS plaque size in both ApoE−/− and Ldlr−/− mice (Figure 1D and Supplementary Figure 4D). In both ApoE^−/−^ and Ldlr^−/−^ mice, the high dose of omentin-1 (5 μg/kg/h) helped to reduce the lipid content in the AS plaque (Figures 1E,F and Supplementary Figures 4E,F).

### Omentin-1 Infusion Reduces Inflammatory Cytokines Expression, Macrophage Apoptosis, and Infiltration in the AS Plaque of ApoE−/− and Ldlr−/− Mice

**Figure 2:**
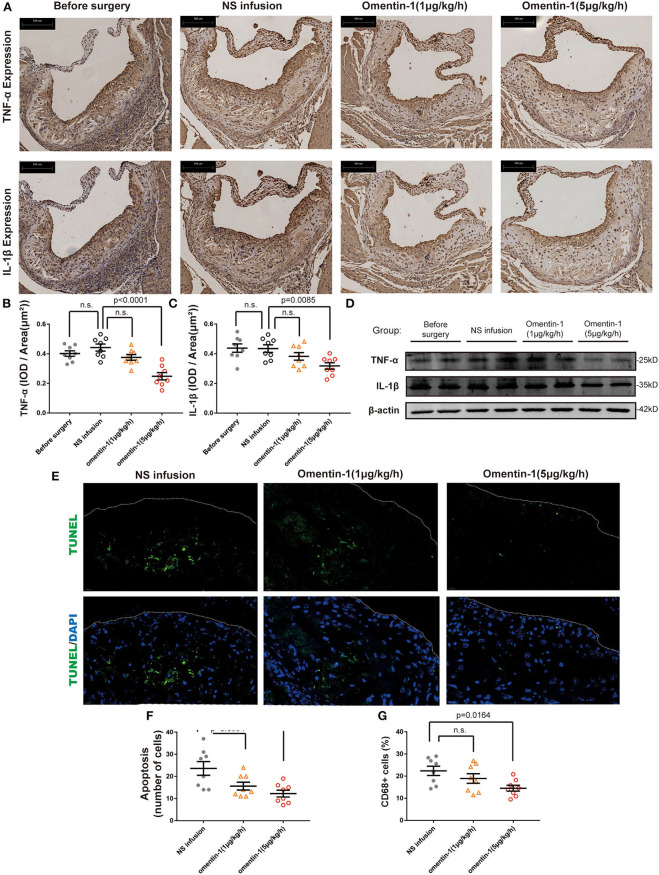
Omentin-1 infusion reduces inflammatory cytokines expression and cell death in atherosclerotic plaque of ApoE^−/−^ mouse. **(A)** Representative micrographs of the aortic root sections of ApoE^−/−^ mice stained by the antibodies against IL-1β or TNF-α. **(B,C)** Graph shows the intensity of inflammatory cytokines expression (n_1,2,3,4_ = 8). The results of immunohistochemistry analysis were expressed in the integral optical density (IOD) of positive staining area vs. plaque area [(IOD, sum)/(Plaque area, μm^2^)]. **(D)** Aortic root samples were collected from animal models and the expression of TNF-α and IL-1β were quantified by western blot analysis. **(E)** Representative images of the aortic root sections of the mouse were stained by DAPI (Abcam, ab104109, displayed in blue) and the apoptotic cell was detected by TUNEL staining (green). The plaque area was marked by a dot line. **(F)** The graph shows the cells undergoing apoptosis in the AS lesion. The intensity of apoptosis was quantified by counting the apoptotic cells (TUNEL+ cells) in each sample (n_1,2,3,4_ = 8). **(G)** The macrophage infiltration in each sample was quantified by calculating the proportion of CD68 positive area (CD68+ area/ total plaque area, n_1,2,3,4_ = 8). All the data in this figure were presented as mean ± SEM [n.s., non-significant; n_1_, the number of subjects in before surgery group; n_2_, the number of subjects in NS infusion group; n_3_, the number of subjects in group infused with omentin-1 (1 μg/kg/h); n_4_, the number of subjects in group infused with omentin-1 (5 μg/kg/h)].

The recruitment of macrophages to the AS lesion is frequently accompanied by the secretion of inflammatory cytokines, which subsequently promoted cell death and increased plaque vulnerability. The mouse IgG isotype was used as a negative control in the immunohistochemical analysis (Supplementary Figure 1C). Here, we examined the inflammatory cytokine expression profile and macrophage infiltration in the ApoE−/− and Ldlr−/− mice (Figure 2A and Supplementary Figure 5A). Statistical analysis revealed that adding omentin-1 to the blood of the mouse significantly alleviated the expression of TNF-α (Figure 2B and Supplementary Figure 5B) and IL-1β (Figure 2C and Supplementary Figure 5C) in the AS lesion. The western blot also indicated that the omentin-1 treatment reduced the TNF-α and IL-1β in the aortic root tissue of the mice (Figure 2D and Supplementary Figure 5D).

The result of the TUNEL staining indicated that either a high or low dose of omentin-1 can reduce the cell apoptosis within the AS plaque in both ApoE−/− and Ldlr−/− mice (Figures 2E,F and Supplementary Figures 5E,F). The antibody against the mouse macrophage marker CD68 was used to investigate the macrophage infiltration in the plaque. Compared with the NS-treated group and the group treated with low dose omentin-1, the macrophage content of the AS lesion is much lower in the high dose omentin-1-treated group in both ApoE−/− and Ldlr−/− mice (Supplementary Figures 2G, 3, 6A,D).

### The Interaction of Omentin-1 With Integrin αvβ3 and αvβ5

**Figure 3:**
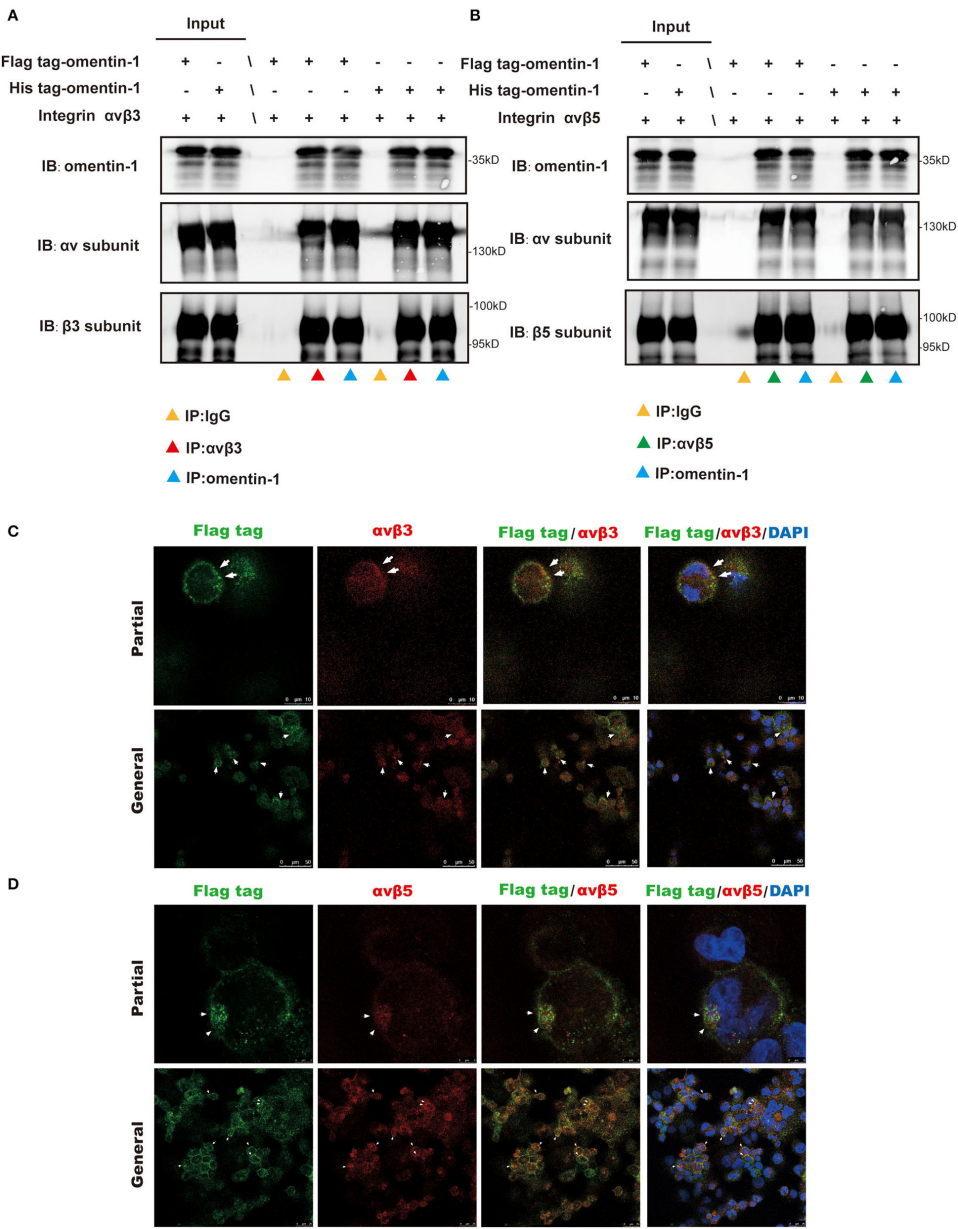
Co-Immunoprecipitation (IP) analysis showed the molecular interaction of omentin-1 (labeled by his tag and flag tag) and integrin αvβ3 and αvβ5. *In vitro* confocal microscopy demonstrated the spatial co-localization of omentin-1 (labeled by flag tag) and integrin receptors on the surface of THP1-derived macrophages. **(A,B)** The results of the co-IP analysis of omentin-1 and integrin receptors, αvβ3 and αvβ5. **(C,D)** *In vitro* confocal microscopy of exogeneous omentin-1 and integrin receptors. The area where exhibited the co-localization of exogenous omentin-1 (green) and integrin receptors (red) was pointed out by white arrows.

To validate the interaction of omentin-1 to the integrin receptor αvβ3 and αvβ5 *in vivo* and *in vitro*, we performed co-immunoprecipitation and immunofluorescence colocalization experiments. The recombinant omentin-1 proteins that have been labeled with his tag or flag tag were, respectively, used to verify their conjugation to integrin αvβ3 and αvβ5. The results of co-immunoprecipitation showed that omentin-1 can bind to the integrin αvβ3 as well as αvβ5, and the adding of artificial peptide tags had no effects on its binding ability (Figures 3A,B).

Furthermore, we applied confocal microscopy to confirm the spatial colocalization of omentin-1 and integrin receptors. The mouse macrophage cell line RAW264.7 was incubated with mild oxidized LDL (ox-LDL) to transform it into a macrophage-derived foam cell. The results of the immunofluorescence staining demonstrated that the cell model of the macrophage-derived foam cell can abundantly express integrin αvβ3 and αvβ5. Besides, after being added to the cell culture solution, the exogenous omentin-1 (labeled by flag tag) exhibited significant colocalization with integrin αvβ3 and αvβ5 on the cell surface in comparison with the IgG isotype (Figures 3C,D and Supplementary Figure 6C).

**Figure 4:**
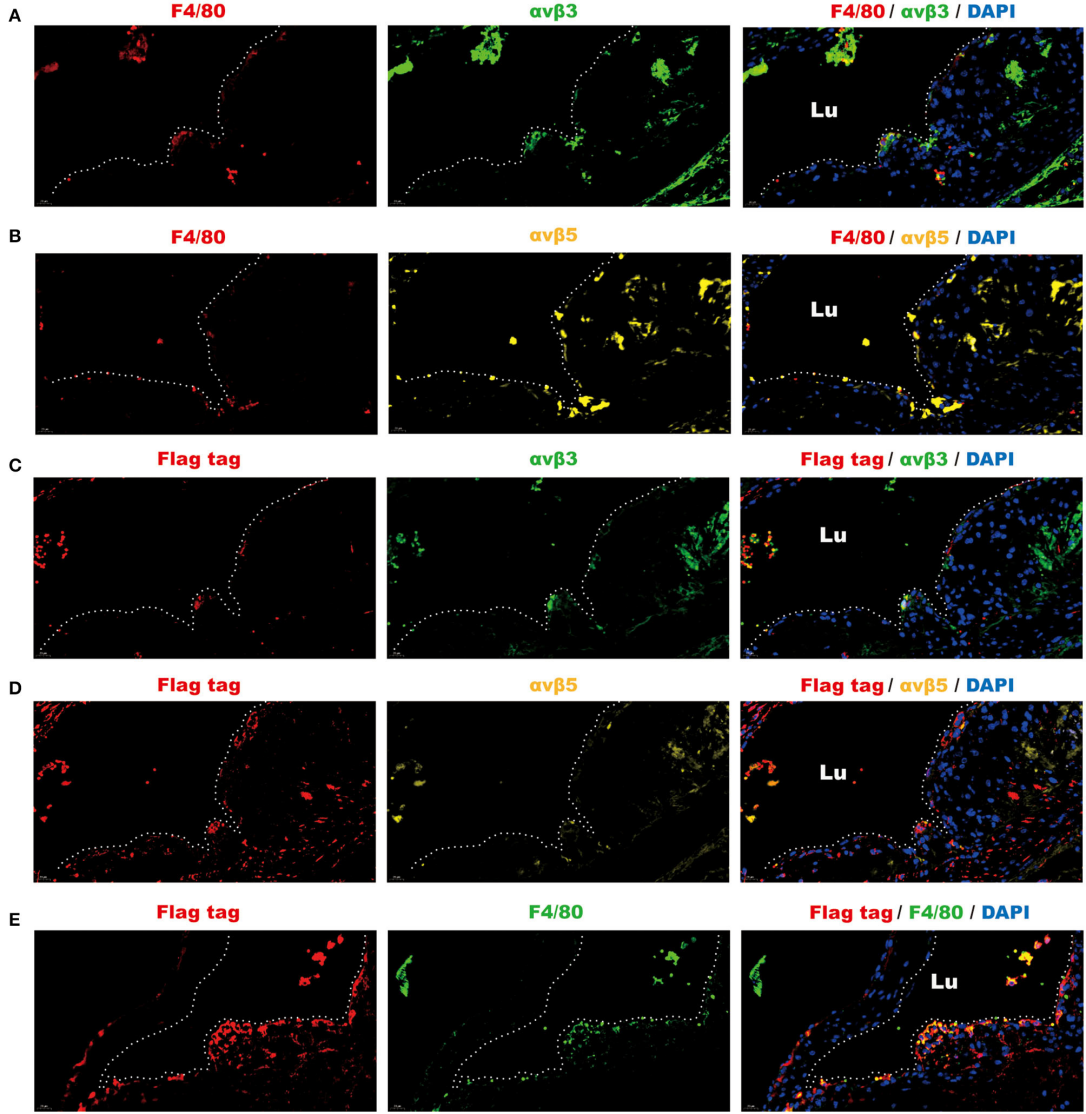
Fluorescence analysis demonstrated the spatial co-localization of infiltrated macrophages, exogenous omentin-1 (labeled by flag tag), and integrin receptors in the atherosclerotic plaque of ApoE^−/−^ mice. The aortic root sections were stained by primary antibodies against F4/80 (macrophage marker), αvβ3, αvβ5, and flag tag (the marker of exogenous omentin-1). Fluorescence secondary antibodies against protein from mice, rabbits, and rats were used to mark the localization of the primary antibodies. Three biological replicates were made in this analysis. **(A)** The macrophage (F4/80) was dyed red and αvβ3 was dyed green. The plaque area was marked by a dotted line. **(B)** The macrophage (F4/80) was dyed red and αvβ5 was dyed yellow. The plaque area was marked by a dotted line. **(C)** Flag-tagged exogenous omentin-1 (flag tag) was dyed red and αvβ3 was dyed green. The plaque area was marked by a dotted line. **(D)** Flag-tagged exogenous omentin-1 (flag tag) was dyed red and αvβ5 was dyed yellow. The plaque area was marked by a dotted line. **(E)** Flag-tagged exogenous omentin-1 (flag tag) was dyed red and macrophage (F4/80) was dyed green. The plaque area was marked by a dotted line (Lu = the lumen of the aorta).

To further validate that the intravenous infusion of the omentin-1-containing solution can lead to the binding of exogenous omentin-1 (labeled by flag tag) to the macrophage integrin receptors in the plaque area of the plaque, we used antibodies against the F4/80, αvβ3, αvβ5, and flag tag to confirm the spatial expression profile of these proteins in the ApoE^−/−^ mouse model. Immunofluorescence confocal microscopy indicated that the mouse macrophage marker F4/80 colocalized with αvβ3 and αvβ5 in the AS lesion (Figures 4A,B), which suggested that the macrophage infiltrating in the plaque can express these two integrin receptors. Moreover, it has also been shown that the exogenous omentin-1 (labeled by flag tag) colocalized with integrin αvβ3 and αvβ5 in the AS lesion (Figures 4C,D), which strongly supported what we had discovered in the co-IP analysis. In addition, we found that exogenous omentin-1 (labeled by flag tag) significantly colocalized with the macrophage marker F4/80 in the plaque site (Figure 4E), which demonstrated that the intravenous infusion of the omentin-1-containing solution can successfully bring omentin-1 to the macrophages that infiltrated the AS lesion. Three biological replicates were made in this analysis (Supplementary Figures 7, 8). The mice infused with NS were used as the control group (Supplementary Figure 9).

**Figure 5:**
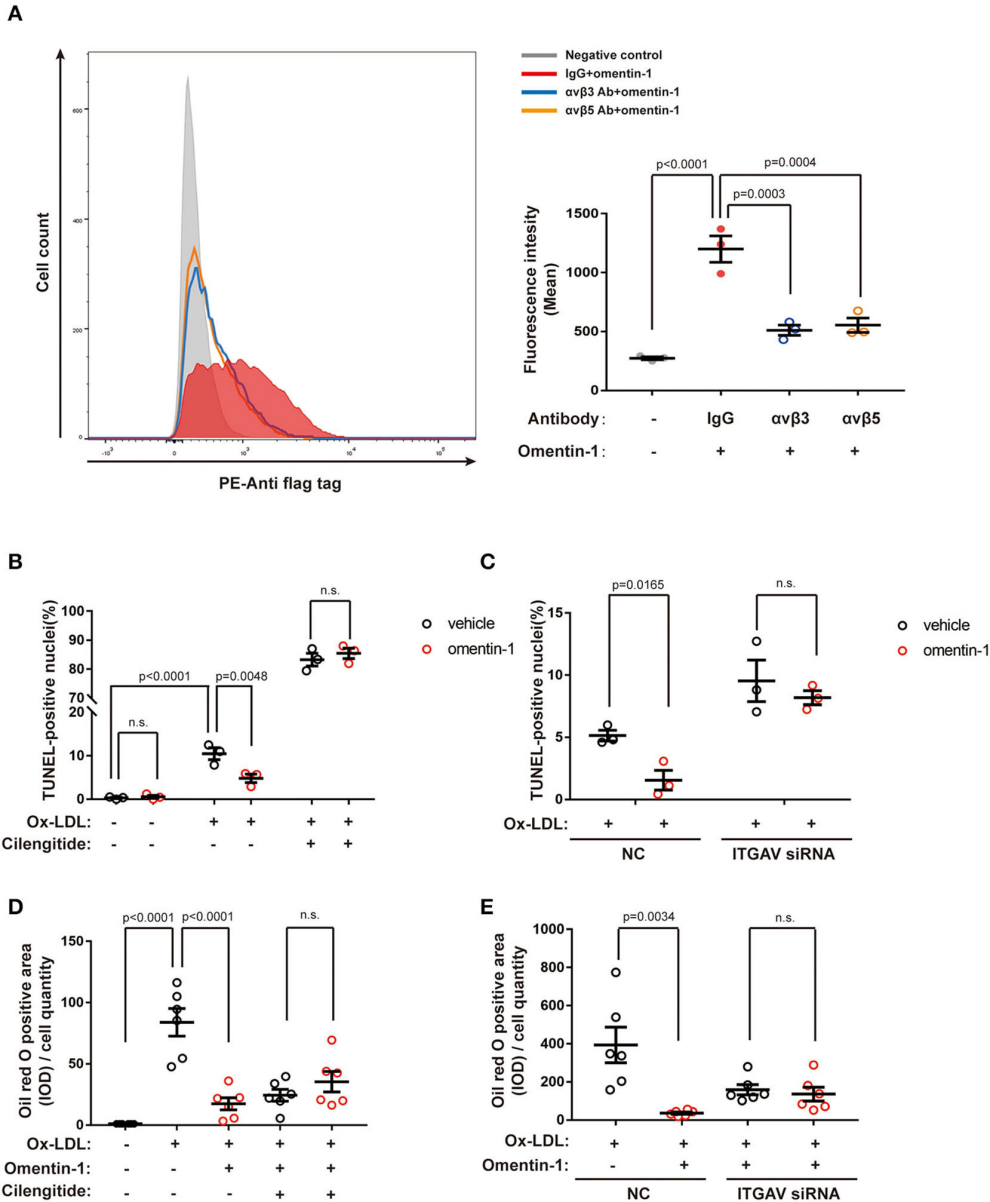
Neutralizing the antibody against the integrin receptor reduced omentin-1 binding to the cell membrane. Omentin-1 attenuated the apoptosis and lipid burden induced by ox-low-density lipoprotein (LDL) in macrophages. **(A)** THP1 cells were induced by 100 ng/ml PMA for 48 h to transform into a macrophage phenotype. After being pretreated by the IgG isotype or neutralizing antibodies against human integrin αvβ3 or αvβ5, the cells were then incubated with Flag-tagged omentin-1. The retention of omentin-1 on the cell membrane was detected by the antibody against the flag tag (PE-labeled). The mean intensity of PE fluorescence was quantified and used to express the magnitude of omentin-1 retention (*n* = 3). **(B)** The RAW264.7-derived macrophages were pretreated by omentin-1 (800 ng/ml) for 1.5 h, and then they were co-incubated with high ox-LDL (50 μg/ml) for 24 h. Apoptosis of macrophages was probed by an *in situ* cell death detection kit (TUNEL), and the apoptotic rate was presented as the percentage of TUNEL-positive nuclei (*n* = 3). **(C)** The RAW264.7-derived macrophages were transfected by ITGAV siRNA to knock down the expression of ITGAV. Then they were incubated with omentin-1 and high ox-LDL to induce apoptosis. The apoptotic rate was assessed as described above (*n* = 3). **(D)** The RAW264.7-derived macrophages were pretreated by omentin-1 (900 ng/ml) for 1.5 h, and then they were co-incubated with mild ox-LDL (50 μg/ml) for 24 h. The lipid content of the cells was detected by an oil red o stain kit. The integral optical density (IOD) of the oil red o positive area vs. cell counts was calculated to represent the lipid retention in cells (*n* = 6). **(E)** The RAW264.7-derived macrophages were transfected by ITGAV siRNA to knock down the expression of ITGAV. Then they were incubated with omentin-1 and mild ox-LDL to induce apoptosis. The lipid retention of cells was assessed as described above (*n* = 6). All data in this figure were presented as mean ± SEM (n.s., non-significant).

Moreover, we used the neutralizing antibodies against αvβ3 or αvβ5 to investigate the competitive effect between integrin ligands and omentin-1 (flag tag labeled) in live cells. The result of flow cytometry indicated that the pre-treatment of neutralizing antibodies impaired the binding of omentin-1 to the cell membrane in the THP-1 cells (Figure 5A).

### Omentin-1 Regulates Apoptosis, Lipid Loading, and Inflammatory Cytokines Expression in Macrophages by Interacting With Integrin Receptors

Our preliminary experiment confirmed that mild ox-LDL could only induce lipid loading instead of apoptosis in macrophages, while high ox-LDL was able to induce inflammation and apoptosis resembling pathological conditions. Therefore, we utilized high ox-LDL to trigger cell apoptosis and stimulate the inflammatory secretion and used mild ox-LDL to induce lipid loading in the macrophages. The integrin subunit αv (ITGAV) siRNA was used to knock down the expression of αv integrin in the RAW264.7 cell line, and the transfection efficiency was examined by RT-qPCR analysis and western blot analysis (Supplementary Figure 14A).

The RAW264.7-derived macrophages were pretreated by omentin-1 for 1.5 h, and subsequently co-incubated with high ox-LDL to induce apoptosis. TUNEL analysis was used to verify the apoptotic rate induced by the high ox-LDL in different groups. The results showed that omentin-1 protected the macrophages from high ox-LDL induced apoptosis, and the blockade of integrin receptor αvβ3 and αvβ5 or knockdown the expression of ITGAV remarkably reversed the anti-apoptosis effect of omentin-1 (Figures 5B,C and Supplementary Figures 10, 11).

The RAW264.7-derived macrophages were treated with omentin-1 and mild ox-LDL to induce lipid loading within the cytoplasm. An oil red O stain kit was used to detect the lipid content of the macrophages. The results showed that omentin-1 remarkably reduced lipid loading in the macrophages. The effect of omentin-1 on combating lipid loading was inhibited upon the ligation of αvβ3 and αvβ5. The knockdown of the αv integrin suppressed the action of omentin-1 on reducing lipid loading (Figures 5D,E and Supplementary Figures 12, 13).

**Figure 6:**
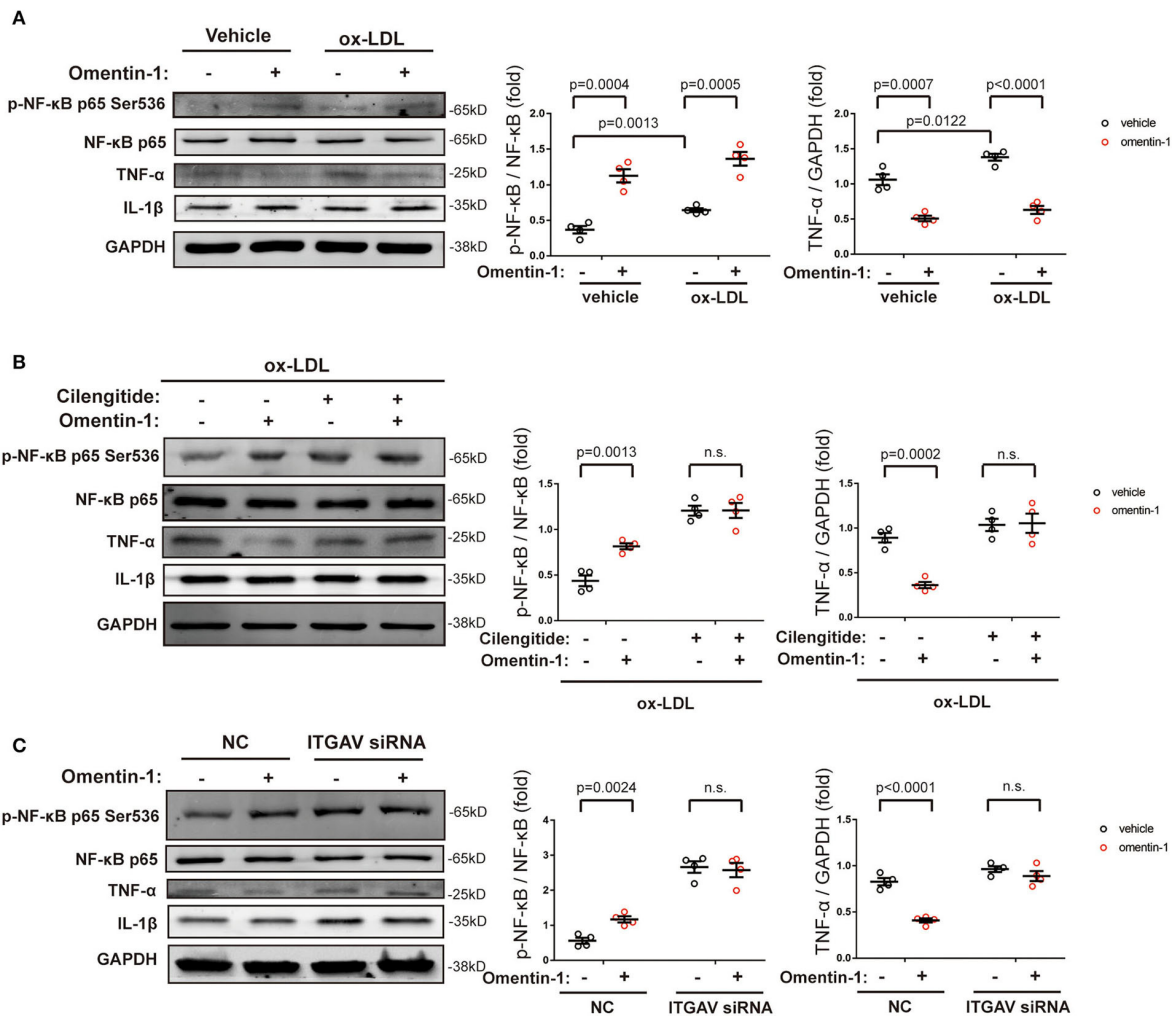
The effects of omentin-1 on inflammatory cytokines expression in the macrophage. **(A)** The RAW264.7-derived macrophages were pretreated by omentin-1 (700 ng/ml) for 1 h. After that, the vehicle (sterilized water) and high ox-LDL (50 μg/ml) were added to the medium and co-incubated with cells for 6 h. The cells were harvested for immunoblotting analysis. The greyscale of each blot was quantified and normalized (*n* = 4). **(B)** The RAW264.7-derived macrophages were pretreated by cilengitide (2.5 μM) for 1 h and co-incubated with or without omentin-1 (700 ng/ml) for another 1 h. After that, they were all stimulated by high ox-LDL (50 μg/ml) for 6 h. The cells were harvested for immunoblotting analysis. The greyscale of each blot was quantified and normalized (*n* = 4). **(C)** The RAW264.7-derived macrophages were transfected by ITGAV siRNA for 6 h. Then the medium was replaced by RPMI 1640 [containing 5% fasting blood sugar (FBS)] and the cells were incubated for 15 h. After that, omentin-1 (700 ng/ml) was added to the medium and co-incubated with cells for 1 h. All of the cells were then treated by high ox-LDL for 6 h. The cells were harvested and subjected to immunoblotting analysis. The greyscale of each blot was quantified and normalized (*n* = 4). All data in this figure were presented as mean ± SEM (n.s., non-significant).

The immunoblotting analysis showed that the high ox-LDL had a significant influence on the NF-κB phosphorylation and TNF-α expression. Upon omentin-1 stimulation, the phosphorylation of NF-κB was enhanced while the TNF-α expression was suppressed (Figure 6A). Upon the inhibition of αvβ3 and αvβ5 by the cilengitide or knockdown of ITGAV by siRNA, the influence exerted by omentin-1 remarkably diminished (Figures 6B,C). The expression of IL-1β was not significantly impacted under the experimental condition.

### Omentin-1 Activates the Downstream Signaling Pathway by Stimulating Integrin Receptors

**Figure 7:**
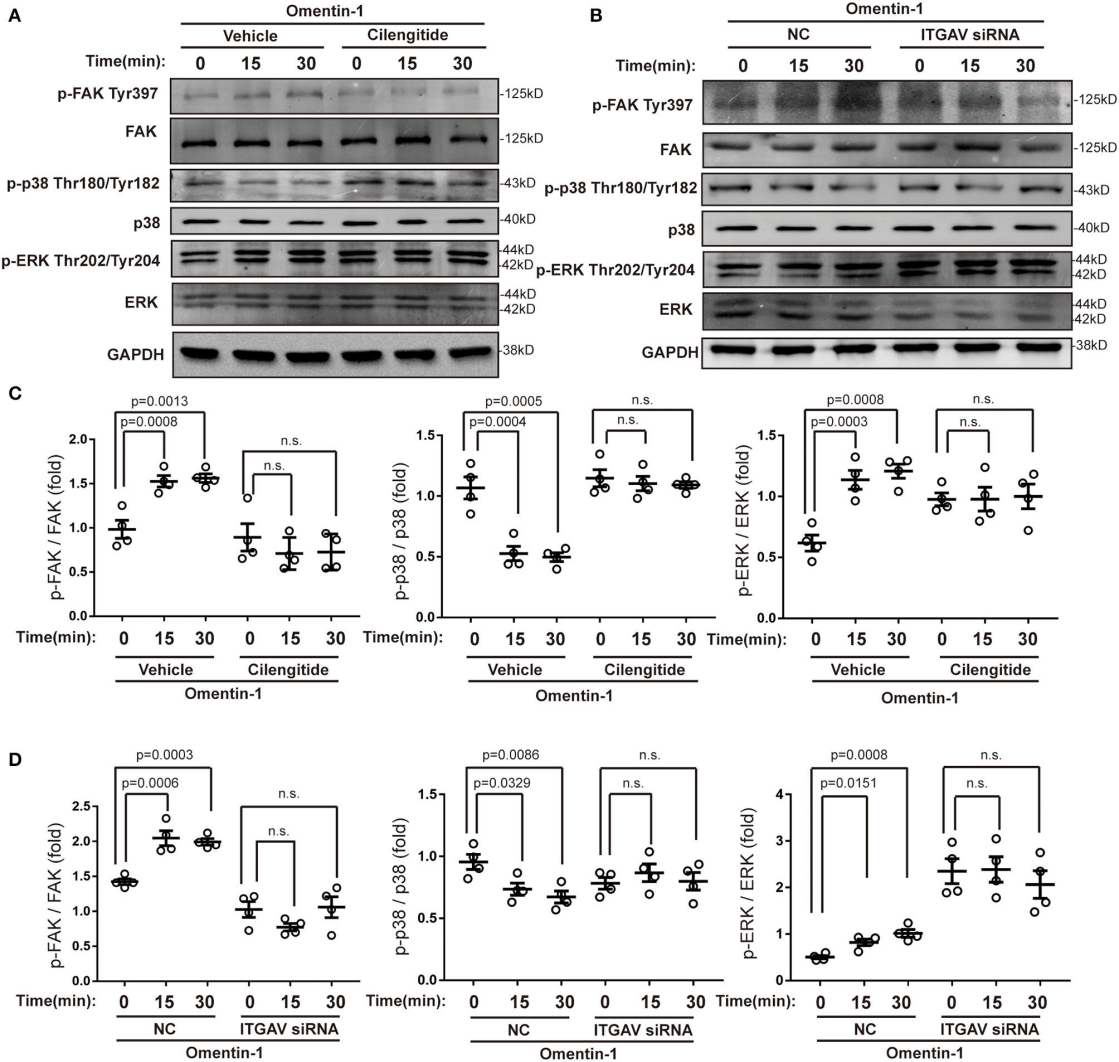
Omentin-1 triggered signals of integrin receptor in macrophages. **(A)** The RAW264.7-derived macrophages were pretreated by vehicle or cilengitide (2.5 μM) for 1 h and were subsequently stimulated by omentin-1 (600 ng/ml) for 0, 15, and 30 min. After that, they were harvested for immunoblotting analysis. **(B)** The RAW264.7-derived macrophages were transfected by negative control (NC) small-interfering RNA (siRNA) or ITGAV siRNA. Then they were stimulated by omentin-1 (600 ng/ml) for 0, 15, and 45 min. After that, they were harvested for immunoblotting analysis. **(C)** The graphs showed that omentin-1 significantly promoted the phosphorylation of focal adhesion kinase (FAK) at Tyr397 and p38 mitogen-activated protein kinase (p38 MAPK) at Thr180/Tyr182, while it suppressed the phosphorylation of the extracellular-regulated protein kinase (ERK) at Thr202/Tyr204 concomitantly. The adding of cilengitide abrogated the influence induced by omentin-1 in the RAW264.7-derived macrophage (*n* = 4). **(D)** The graphs showed that omentin-1 significantly promoted the phosphorylation of FAK at Tyr397, p38 MAPK at Thr180/Tyr182, and suppressed the phosphorylation of ERK at Thr202/Tyr204 in the RAW264.7-derived macrophage transfected by NC siRNA. After being transfected by ITGAV siRNA, the influence induced by omentin-1 was abrogated (*n* = 4). All the data in this figure were presented as mean ± SEM (n.s., non-significant).

To validate that the integrin αvβ3 and αvβ5 are functional receptors of omentin-1, we also examined the phosphorylation status of the downstream signaling pathway of the integrin receptor. Immunoblotting analysis indicated that omentin-1 significantly promoted the activation of focal adhesion kinase (FAK) and extracellular-regulated protein kinase (ERK). On the contrary, omentin-1 reduced the phosphorylation of p38 mitogen-activated protein kinase (p38 MAPK). The ligation of integrins by cilengitide and the knockdown of ITGAV both reversed the phosphorylation of the signaling pathway induced by omentin-1 (Figure 7).

Ras-related C3 botulinum toxin substrate 1 (Rac1) is a member of the Rho GTPase family, which is frequently activated by integrin signals. We also examined the activity of Rac1 by assessing its membrane translocation. The membrane protein and cytosol protein were extracted, respectively, and the Rac1 expression was measured by immunoblotting analysis. The results showed that omentin-1 promoted the translocation of Rac1 from the cytoplasm to the membrane, and this process could be inhibited by cilengitide (Supplementary Figure 14B).

**Figure 8:**
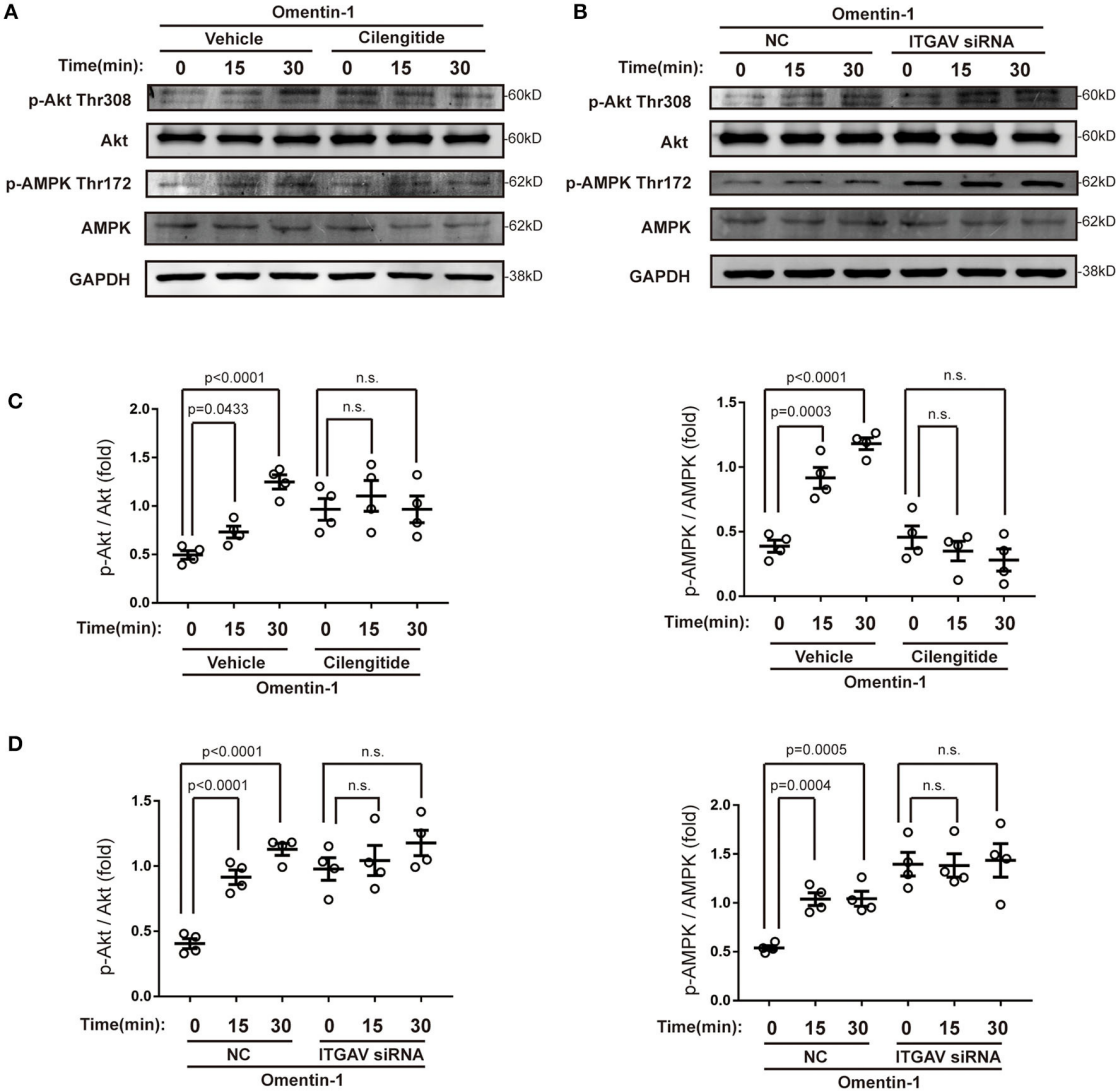
Omentin-1 promoted the phosphorylation of Akt and AMPK *via* integrin receptor. **(A)** The RAW264.7-derived macrophages were pretreated by vehicle or cilengitide (2.5 μM) for 1 h and were subsequently stimulated by omentin-1 (600 ng/ml) for 0, 15, and 30 min. After that, they were harvested for immunoblotting analysis. **(B)** The RAW264.7-derived macrophages were transfected by NC siRNA or ITGAV siRNA. Then they were stimulated by omentin-1 (600 ng/ml) for 0, 15, and 45 min. After that, they were harvested for immunoblotting analysis. **(C)** Graphs showed that omentin-1 significantly promoted the phosphorylation of Akt at Thr308 and AMPK at Thr172. The adding of cilengitide abrogated the influence induced by omentin-1 in the RAW264.7-derived macrophage (*n* = 4). **(D)** Graphs showed that omentin-1 significantly promoted phosphorylation of Akt at Thr308 and AMPK at Thr172 in the RAW264.7-derived macrophage transfected by NC siRNA. After being transfected by ITGAV siRNA, the influence induced by omentin-1 was abrogated (*n* = 4). All the data in this figure were presented as mean ± SEM (n.s., non-significant).

Several previous studies have demonstrated that omentin-1 induces its biological effects *via* adenosine 5′monophosphate-activated protein kinase (AMPK) and the Akt pathway. In this study, we also investigated the effect of omentin-1 on AMPK and Akt phosphorylation in the RAW264.7 macrophage model. The results of the immunoblotting showed that omentin-1 promoted the phosphorylation of both AMPK and Akt. Both the use of cilengitide and the knockdown of ITGAV mitigated the effect of omentin-1 on AMPK and Akt phosphorylation (Figure 8).

## Discussion

Previous pathological studies of AS lesions from patients with ACS indicated that plaque rupture, which was the sign of vulnerable plaque, was linked to the formation of thin-cap fibroatheroma (TCFA) (15, 16). The increase of plaque collagen content, coupled with the reduction of necrotic cores and macrophage infiltration, was proved to be associated with the reduction of TCFA (17, 18). In this research, we investigated the effects induced by omentin-1 on the AS plaque vulnerability. In both ApoE^−/−^ and Ldlr^−/−^ mice with already-established AS lesions, the systemic delivery of human omentin-1 significantly increased the collagen content and fibrous cap thickness, mitigated the formation of the necrotic core, and suppressed the expression of inflammatory cytokines within the plaque. Furthermore, omentin-1 modulated the macrophage viability and inflammation status by interacting with the integrin receptor αvβ3 and αvβ5 *in vitro*. These data suggest that omentin-1 acts as an anti-inflammatory adipokine that can enhance the stability of the AS plaque by modulating the macrophage function *via* the integrin receptor αvβ3 and αvβ5.

Recent studies indicated that adipose tissue not only functions as an energy storage organ but is also involved in endocrine regulation (19). Adipokines extensively participate in various physiological processes (20). Du et al. demonstrated that omentin-1 expression is lower in the EAT adjacent to the coronary stenotic segments than the non-stenotic segments (7). Watanabe et al. and Hiramatsu-Ito et al. revealed that omentin-1 had counteractive effects on atherogenesis (10, 21). But whether omentin-1 can regulate the stability of already-established plaque remain to be studied. In addition, Yoshiyuki et al. revealed that omentin-1 can protect the myocardium from ischemia-reperfusion injury by activating Akt and AMPK, but the receptor of omentin-1 has not been identified yet (22).

To investigate the therapeutic value of omentin-1, we decided to inject an omentin-1 solution into the jugular vein of a mouse with an already-established AS plaque. We found that after 3 weeks of treatment with omentin-1, the AS plaque exhibited reduced necrotic cores formation and macrophage infiltration, while the collagen content increased significantly. The changes in these three indices were linked to the elevation of plaque stability. Moreover, after the treatment with omentin-1, the expression of TNF-α and IL-1β were significantly suppressed in the AS plaque of the mice. Taken together, all these findings from the animal experiments suggested that omentin-1 treatment can attenuate the vulnerability of already-established plaque, and has a potential applicable value under clinical conditions.

Integrin receptors are expressed in almost all cell types, and they play an important role in cellular crosstalk with its microenvironment. As a transmembrane receptor, integrin serves to integrate the ECM with the internal actomyosin cytoskeleton and alter the cell adhesion and transduce viability signals concomitantly. Integrin αvβ3, which was used to be a marker of tissue repairing and angiogenesis, is now considered to be a promising biomarker of vulnerable plaque (23). The ligation of αvβ3 was proved to be an effective way of attenuating coronary artery atherosclerosis *in vivo* (24). Hoshiga et al. demonstrated that the expression of αvβ3 is higher in AS vessels compared with non-AS vessels. These findings suggest that integrin receptors may have an underlying association with the development of AS. Moreover, in their study, they also found that the expression of αvβ3 is prominent in the adventitia, in contrast to a much lower expression level of it in percutaneous similar-sized microvessels (25). Integrin αvβ3 and omentin-1 shared similar expression profiles in humans: they are primarily expressed by visceral vessels (or adipose tissue) instead of subcutaneous vessels (or adipose tissue). Tsuji et al. discovered that omentin-1 (intelectin-1) contained a fibrinogen-like domain, this discovery suggested that omentin-1 was a potential ligand of integrin (26). All these findings inspired us to further investigate the relationship between omentin-1, integrin receptors, and atherosclerosis.

Macrophages are considered to be closely associated with the progression and vulnerability of the AS plaque. In our study, we discovered that omentin-1 exhibited abundant co-localization with macrophage in the plaque site. Confocal analysis performed *in vivo* and *in vitro* indicated that omentin-1 significantly co-localized with the integrin receptor αvβ3 and αvβ5. Using co-IP analysis, we confirmed the molecular interaction between omentin-1 and integrin receptor, αvβ3 and αvβ5. Through immunoblotting analysis, we discovered that omentin-1 enhanced the phosphorylation FAK, ERK, Akt, and AMPK, while it reduced the phosphorylation of p38 MAPK. Previous studies attributed most of the biological effects of omentin-1 to the activation of Akt and AMPK (27). The study conducted by Yoshiyuki et al. found that the pretreatment with αvβ3 antibody suppressed the omentin-1-induced increase in the Akt phosphorylation, whereas it did not affect the omentin-induced AMPK activation (22). In our research, we discovered that omentin-1 can bind to both αvβ3 and αvβ5, and the pretreatment with cilengitide or knockdown of ITGAV can mitigate the effect of omentin-1 on AMPK and Akt phosphorylation. Our findings are consistent with previous studies that demonstrated that while the phosphorylation of Akt can be induced *via* αvβ3, the phosphorylation of AMPK can be induced by the activation of αvβ5 (28). Protein Rac1, which is a member of the Rho GTPase family, is frequently stimulated by integrin signals (29). The translocation of Rac1 from the cytoplasm to the membrane is required for the initiation of the activity of Rac1 (30). In our study, we observed the significant membrane translocation of Rac1 upon omentin-1 stimulation, which further proved that integrin receptors serve as the functional receptor of omentin-1. After the adding of cilengitide (potent blocker of αvβ3 and αvβ5) or knocking-down the expression of ITGAV (the common subunit of αvβ3 and αvβ5), the signals transduced by omentin-1 were significantly inhibited. This finding demonstrated that αvβ3 and αvβ5 played an indispensable role in transducing the signals of omentin-1. Additionally, through the flow cytometry analysis, we discovered that the binding of omentin-1 to the cell membrane can be reduced by the antibodies against αvβ3 or αvβ5 which further demonstrated that the biological effect induced by omentin-1 could be regulated by other integrin ligands.

The engulfment of ox-LDL tends to activate macrophages, which subsequently induces the secretion of inflammatory cytokines, cell apoptosis, and eventually contributes to the formation of vulnerable AS plaques (31). In this study, we testified that omentin-1 reduced cell apoptosis and lipid loading mainly by interacting with αvβ3 and αvβ5. Animal studies indicated that the intravenous infusion of omentin-1 remarkably reduced the expression of TNF-α and IL-1β in the AS plaque. Cell experiments revealed that omentin-1 treatment only significantly attenuated the TNF-α expression induced by high ox-LDL, whereas the expression of IL-1β was not impacted. The explanation for these rather contradictory results may be that the activation of integrin receptors inhibited the cell apoptosis and necrotic core formation in the AS lesion, and the reduction in cell debris, in turn, alleviated the inflammatory response in the plaque (4). Moreover, we also discovered that omentin-1, which has long been considered an anti-inflammation adipokine, had a stimulatory effect on NF-κB p65 phosphorylation. Although the phosphorylation of NF-κB p65 on ser536 was considered to promote the transcription of inflammatory cytokines, our study indicated that the phosphorylation of NF-κB did not impact the anti-inflammation function of omentin-1. However, our research also indicated that omentin-1 inhibited the phosphorylation of p38 MAPK, which was a positive regulator of TNF-α (32). The inhibition of p38 MAPK phosphorylation, which was induced by omentin-1, may be a plausible explanation for this result.

Our data revealed that omentin-1 produced favorable effects on plaque stability by interacting with αvβ3 and αvβ5 integrins in macrophage. Extracellular matrix proteins, such as fibronectin, vitronectin, and fibrinogen, bind to integrin receptors and elicit downstream signals (outside-in signaling). Several studies have reported that the activation of integrin receptors was associated with the increase of the resistance to cell apoptosis (33). The ligand-bound integrins regulate the actin network through additional adaptor proteins, leading to integrin clustering and the activation of FAK and the Src family kinase. Upon the assistance of FAK, Src phosphorylates downstream substrates, including ERK and PI3K/Akt pathways. These pathways subsequently produce pro-survival effects by promoting the phosphorylation of cytoplasmic targets (34).

Our findings provided a key explanation for previous studies focused on omentin-1. Yin et al. reported that omentin-1 exerted beneficial effects on mesenchymal stem cells by inhibiting apoptosis, promoting proliferation, and increasing the secretion of angiogenic cytokines (35). These functions are consistent with the bioeffects induced by integrin receptors (36). Besides, Antonov AS et al. reported that the ligation of the integrin receptors also prevented the transformation of blood monocyte and macrophage into foam cell phenotype by the down-regulation of CD36 and scavenge receptor-A, which supported the results found by Hiramatsu-Ito et al. (21, 37).

Integrins are widely implicated in cell adhesion, proliferation, phagocytosis, and vascular formation. Several studies have explored that integrins played a vital role in atherosclerosis and the metabolic process (38, 39). The bioinformatic analysis of the EAT of coronary artery disease (CAD) patients revealed that the focal adhesion pathway, which was mainly a downstream signal of integrin receptors, may act a significant role in coronary AS pathogenesis (40). Because omentin-1 has exhibited a unique protective effect on the cardiovascular system, a detailed analysis of the omentin-1 structure and its biological active motif is needed to be done in the future. Furthermore, our research also suggests that the integrin receptor αvβ3 and αvβ5, as the receptor of omentin-1, are a potential target in treating cardiovascular diseases.

## Data Availability Statement

The raw data supporting the conclusions of this article will be made available by the authors, without undue reservation.

## Ethics Statement

The animal study was reviewed and approved by Capital Medical University Animal Care and Use Committee.

## Author Contributions

XL is the first author of this study. XL, YS, and ZW conceived the experiments and conducted the experiments. XL drafted the manuscript. SY, MY, LP, JieY, JiaY, QS, JL, YL, and YZ helped to analyze and interpret the data. All authors read and approved the final manuscript.

## Funding

This study was supported by a grant from the National Natural Science Foundation of China (81670391).

## Conflict of Interest

The authors declare that the research was conducted in the absence of any commercial or financial relationships that could be construed as a potential conflict of interest.

## Publisher's Note

All claims expressed in this article are solely those of the authors and do not necessarily represent those of their affiliated organizations, or those of the publisher, the editors and the reviewers. Any product that may be evaluated in this article, or claim that may be made by its manufacturer, is not guaranteed or endorsed by the publisher.

## Abbreviations

ACS: acute coronary syndrome
Akt: protein kinase B
AMPK: adenosine 5‘-monophosphate-activated protein kinase
ApoE−/− mice: apolipoprotein E-deficient mice
AS: atherosclerosis
EAT: epicardial adipose tissue
ERK: extracellular-regulated protein kinase
FAK: focal adhesion kinase
HDL: high-density lipoprotein
ITGAV: integrin subunit αv
LDL: low-density lipoprotein
Ldlr−/− mice: low density lipoprotein receptor-deficient mice
NS: normal saline
ox-LDL: oxidized low-density lipoprotein
p38 MAPK: p38 mitogen-activated protein kinase
PI3K: phosphatidylinositol 3-kinase
Rac1: ras-related C3 botulinum toxin substrate 1
siRNA: small interfering RNA
TCFA: thin-cap fibroatheroma.
