# Supplementary material for: Omentin-1 Modulates Macrophage Function via Integrin Receptors αvβ3 and αvβ5 and Reverses Plaque Vulnerability in Animal Models of Atherosclerosis
Source: Front Cardiovasc Med. 2021 Nov 2;8:757926. doi: 10.3389/fcvm.2021.757926 (PMC8593239; doi:10.3389/fcvm.2021.757926)
Supplement: Supplementary file 1 [file Data_Sheet_1.docx]

**Supplemental material**

**1.Study methods**

**1.1Animal model:** Animal experiments were performed in accordance with Institutional Animal Care and Use of Laboratory Animals and were approved by the Capital Medical University Animal Care and Use Committee. And all of the animal experiments performed by us were in conformity with European Community guiding principles in the Care and Use of Animals (2010/63/UE). A total of 58 male ApoE^-/-^ mouse (C57BL/6 background) at the age of 8 weeks were purchased from Beijing Vital River Laboratory Animal Technology. And a total of 40 male Ldlr^-/-^ mouse (C57BL/6 background) at the age of 10 weeks were purchased from Guangzhou Cyagen Biosciences Inc. ApoE^-/-^ mouse were fed on western diet for 12 weeks and Ldlr^-/-^ mouse were fed on western diet for 8 weeks in order to build up atherosclerotic lesion. The groups without minipump implantation were euthanized under deep anesthesia (induced by intraperitoneal injection with an overdose of pentobarbital sodium (200mg/kg)) in order to get their aortas and blood samples. The rest of mouse were randomly divided into 3 groups: (i) controls (infused with normal saline), (ii) low dose omentin-1-treated group (infused with omentin-1 in the rate of 1μg/kg/h *via* ALZET minipump), (iii) high dose omentin-1-treated group (infused with omentin-1 in the rate of 5μg/kg/h *via* ALZET minipump). ALZET minipumps (ALZET Model 2004; Cupertino, CA, USA) were used to achieve continuous infusion of flag-tagged omentin-1 (Abcam, ab157030) solution into mouse jugular vein. For implanting minipumps, anesthesia of mouse was induced by inhaling 2% isoflurane and maintained by inhaling 1.5% isoflurane. Once anethetised, mice was instrumented with a polyurethane catheter (ALZET, Cat No: 0007700; Cupertino, CA, USA) implanted in the right jugular vein. The catheter was attached to a ALZET minipump, which was implanted subcutaneously. All of the remaining mouse were fed on western diet after the implantation of minipumps. After 3-week of infusion, their blood pressure and body weight were measured before receiving euthanasia under deep anesthesia (the method was descripted as above). The whole aorta sample of every mice was obtained and used for immunochemistry analysis and immunofluorescence analysis. The blood sample and whole aorta sample of every mice was obtained. Fasting plasma glucose, triglyceride, total cholesterol, low-density lipoprotein (LDL) cholesterol and high-density (HDL) cholesterol were measured enzymatic methods. The omentin-1 concentration of plasma was measure by ELISA analysis (Biovendor, RD191100200R).

**1.2Mouse aortic root atherosclerotic lesion assessment:** Eight micrometer (8mm) cryosection were taken from the entire region of the valve leaflet and every 10th section (80μm) was subjected to Oil red O staining (Solarbio Life Science, G1262) and hematoxylin counterstaining. Images were captured under identical microscope. Light conditions and camera were NIKON CI-8 and NIKON DS-U3. All of sections were coded and analyzed blind. The value of lipid content (the proportion of oil red O positive area in plaque) of each section was calculated and averaged to obtain the mean lipid content of AS lesion per mouse. Three sections from different levels of aortic root in each mouse were obtained and subjected to HE staining and Masson’s trichrome staining (Solarbio Life Science, G1340). Plaque stability was evaluated by assessing the necrotic cores formation (necrosis area versus total plaque area) and collagen content (proportion of collagen fiber in plaque) of the AS lesion.

Macrophage infiltration was detected by using an antibody against CD68 (Cell Signaling Technology, 97778S), the macrophage content of plaque was expressed as the proportion of CD68+ cells. To determine the pro-inflammatory cytokines expression within the atherosclerotic lesion, immunohistochemistry analysis and western blot were performed by using antibodies against TNF-α (Abcam, ab9739), and IL-1β (Abcam, ab9722). The results of immunohistochemistry analysis were expressed in integral optical density (IOD) of positive staining area versus AS lesion area ((IOD, sum)/ (Plaque area, μm^2^)). To assess integrin αvβ3 and αvβ5 expression of the infiltrated macrophages in the plaque, we performed immunofluorescence experiments. For the purpose of detecting the colocalization of exogenous omentin-1 to the plaque integrin αvβ3 and αvβ5, immunofluorescence co-localization analysis was performed by applying antibodies against flag tag (Abcam, ab205606), integrin αvβ3 (Santa Cruz, sc-7312), integrin αvβ5 (Santa Cruz, sc-13588).

Cell apoptosis in plaque was detected by using *in situ* cell death detection kit (TUNEL assay kit, Roche), and the cells undergoing apoptosis were quantified by counting TUNEL+ cells.

About Six micrometer (6mm) of aortic root tissue was taken from the subjects for western blot analysis. After removing the adherent tissue from the vessel, each sample was cut into pieces. The weight of sample was measured, and lysis buffer (containing 1Mm PMSF) was added to the sample at the ratio of 100μl:20mg. Each of sample was grinded and centrifugated. The supernatant of sample was collected, and protein concentration of each sample was quantified and equilibrated before undergoing western blotting.

**1.3Cell culture and reagents:** RAW264.7 and THP1 cell lines were purchased from Cyagen Bioscience (Guangzhou, China). RAW264.7 and THP1 cells were cultured in RPMI 1640 medium supplemented with 10% fetal bovine serum and 1% penicillin-streptomycin solution. THP1 cells were incubated with PMA (Sigma-aldrich, P1585) containing medium in a concentration of 100ng/ml for 48 hours in order to differentiate them into THP1-derived macrophages. To establish foam cell models *in vitro*, RAW264.7-derived macrophages were incubated with human mild oxidized low-density lipoprotein (ox-LDL) (Yiyuan Biotech, Guangzhou, YB-002) or human high ox-LDL (Yiyuan Biotech, Guangzhou, high ox-LDL) in a concentration of 50μg/ml for 24 hours. Lipid peroxidation was quantified by thiobarbituric acid reactive substances (TBARS) assay. The TBARS of ox-LDL was determined colorimetrically by using Malondialdehyde (MDA) as a standard. The TBARS of mild ox-LDL surpassed 18.5 nmoles of MDA/mg protein (starting LDL was less than 0.50 nmoles of MDA/mg protein), and TBARS of high ox-LDL surpassed 90 nmoles of MDA/mg protein (starting LDL was less than 1 nmoles of MDA/mg protein). After the incubation, the accumulation of lipid droplets within the cytoplasm were detected by using oil red o stain kit. To assess the effects of omentin-1 on foam cell formation, human recombinant omentin-1 (Abcam, ab157030) was added to the culture medium before incubating with ox-LDL.

The small interfering RNA (siRNA) of ITGAV was bought from GenePharma Co., Ltd (Suzhou, China), and the nucleotide sequence is shown in Table 1. The transfection experiment was performed according to the procedure provided by the supplier, the transfection efficiency was determined by RT-qPCR analysis. To further validate the role played by integrin in transducing omentin-1 signals, we also purchased cilengitide, a potent inhibitor to integrin αvβ3 and αvβ5, from MedChemExpress.

**1.4Immunoprecipitation:** To verify the binding of omentin-1 to integrin αvβ3 and αvβ5, we performed immunoprecipitation experiments *in vitro*. Human recombinant omentin-1 protein labeled by flag tag (Abcam, ab157030) and his-tag (Abcam, ab207144) were used to ascertain that the artificial synthetic tags of recombinant omentin-1 were not implicated in the molecule interaction. Recombinant human integrin αvβ3 and αvβ5 protein were purchased from R&D Systems. The antibodies against αvβ3 (R&D Systems, MAB3050), αvβ5 (R&D Systems, MAB2528) and omentin-1 (Proteintech, 11770-1-AP) were used in immunoprecipitation analysis.

Flag-tagged or his-tagged omentin-1 (both of 50ng) was incubated with 100ng of αvβ3 or αvβ5 protein in 0.5ml of buffer containing 50 mM Tris (pH 7.4), 150 mM NaCl, 10% glycerol, and 1 mM DTT for 12 hour at 4°C, then 5μg anti-omentin-1, anti-αvβ3 or anti-αvβ5 antibody was added to each sample and incubated overnight at 4°C. The samples were incubated for additional 2 h with 5μl Sepharose beads. The immunocomplexes were resolved by 12% SDS-PAGE and analyzed by Western blotting using anti-omentin-1 (Proteintech, 11770-1-AP), anti-αv (Proteintech, 27096-1-AP), anti-β3 (Proteintech, 18309-1-AP) or anti-β5 antibodies (28543-1-AP).

**1.5Confocal microscopy:** RAW264.7-derived macrophages were grown in confocal dishes, and they were co-incubated with recombinant omentin-1 (flag tag labeled, Abcam, ab157030) for 2 hours. Then the cells were rinsed by PBS and fixed by 4% Paraformaldehyde. Antibodies against flag tag, integrin αvβ3 (Santa Cruz, sc-7312) and αvβ5 (Santa Cruz, sc-13588) were used to confirm the co-localization of exogenous omentin-1 and integrin receptors *in vitro*. Confocal microscopy was performed by using LEICA TCS SP5 MP.

**1.6Flowcytometry assay:** To validate whether using of neutralizing antibodies against αvβ3 or αvβ5 can impact the binding of omentin-1 to cell membrane, we conducted flowcytometry analysis. 5×10^4^ of THP1 cells were induced by incubating with 100ng/ml PMA for 48 hours in order to differentiate them into THP1-derived macrophages. After that, they were washed by PBS twice and incubated with 16μg/ml of αvβ3-antibody (Abcam, ab190147), αvβ5-antibody (Abcam, ab177004) or IgG isotype (with PBS containing 0.5% FBS) for 1 hour in 37℃. The cells were then washed twice by 0.5%FBS/PBS and further incubated with 1.6μg/ml flag-tagged omentin-1 (Abcam, ab157030) for 45 minutes in 37℃. After incubating, the cells were washed by 0.5%FBS/PBS and labeled by PE Anti-DDDDK (flag) tag (Abcam, ab72469, 1:500 in 0.5%FBS/PBS) for 30 minutes in 37℃. All of cells were washed by 0.5%FBS/PBS twice and PBS once and collected by using enzyme-free cell dissociation buffer (Thermofisher, 13151014). The cell only stained by PE Anti-DDDDK (flag) tag was considered as negative control. Fluorescence intensity of cells was quantified by BD FACSCanto II. The result was analyzed by Flowjo Vx 0.7.

**1.7Western blot:** Western blot experiments were conducted to determine the deviation of protein expression between different groups. Antibodies were purchased from Abcam and Cell Signaling Technology. To assess the translocation of protein rac1 to the membrane, we extracted membrane protein and cytosol protein respectively. Protein concentration of each sample was equilibrated and immunoblotting experiments were conducted in accordance with the procedure. We replicated each experiment for at least 3 times to validate the results we got. We used β-actin and glyceraldehyde-3-phosphate dehydrogenase (GAPDH) to normalized the expression of target protein in each sample.

**1.8RT-qPCR analysis:** TRIzol reagent (Invitrogen) was used to extract total RNAs of cells according to the manufacturer’s guidance. TranScript One-Step gDNA Removal and cDNA synthesis kit (Trasgen Biotech, Beijing, China) was used to reverse transcript isolated RNA to cDNA. RNA expression levels were detected by SYBR Supermix (Transgen Biotech) and 7500 FAST Real-time PCR System (Applied Biosystems, Foster, CA, USA). We used GAPDH to standardized the relative expression of ITGAV gene. The difference of Cq value of GAPDH in each group is less than 1.0. We repeated each sample 2 times and replicated each experiment using at least 4 biological samples. The primer sequence used for RT-qPCR is shown in Table 2.

**1.9Apoptosis assay in vitro:** *In situ* cell death detection kit ((TUNEL assay kit, Roche) was used to determine the apoptotic rate of macrophages *in vitro*. The experiment was conducted under the guidance provided by the manufacturer.

**1.10Oil red o staining in vitro:** The oil red o stain kit (Solarbio, G1262) was used to detect lipid retention in cell and tissue. Samples were processed in accordance with manufacturer’s guidance.

**1.11Statistical analysis:** Results were presented as mean ± SEM for continuous data and as frequencies for categorical data. Unpaired two-tailed Student t test was used for comparison between two groups. One-way ANOVA followed by Bonferroni multiple comparisons post-test was performed to assess the significance of deviation when three or more groups were involved. The χ^2^ test was used to compare the categorial data. All data were analyzed by GraphPad Prism 7 and SPSS 26. P values <0.05 was considered as statistical significance. P values were given in the figures. Representative images were selected as those that show values close to the means of the results obtained from all analyzed samples.

**2.Supplementary figures:**

**Figure S1: Infusion of omentin-1 elevated exogenous omentin-1 retention in plaque of ApoE^-/-^ mouse.** A. Anti-flag tag showed the localization of exogenous omentin-1 (red) in the plaque area of ApoE^-/-^ mouse. The magnitude of flag-tagged omentin-1 retention in plaque was expressed by calculating the proportion of red fluorescence positive area. B. Graph showed that intravenous infusion significantly elevated omentin-1 retention in plaque site. Plaque area was marked by dot line (n=6). (Lu=the lumen of aorta) C. Aortic root sections were stained by mouse IgG isotype, which was used as negative controls in immunohistochemical analysis.

**Figure S2: Infusion of omentin-1 elevated exogenous omentin-1 retention in plaque of Ldlr^-/-^ mouse.** A. Anti-flag tag showed the localization of exogenous omentin-1 (red) in the plaque area of Ldlr^-/-^ mouse. The magnitude of flag-tagged omentin-1 retention in plaque was expressed by calculating the proportion of red fluorescence positive area. B. Graph showed that intravenous infusion significantly elevated omentin-1 retention in plaque site. Plaque area was marked by dot line (n=6). (Lu=the lumen of aorta)

**Figure S3:** **Infusion of omentin-1 reduced macrophage infiltration in plaque of ApoE^-/-^ mouse.** Representative images of mouse’s aortic root sections were stained by DAPI (blue) and the antibody of macrophage marker CD68 (red). The plaque area was marked by dot line, and the red fluorescence area within the atherosclerotic plaque represents the area of macrophage infiltration. The macrophage infiltration in each sample was quantified by calculating proportion of CD68 positive area (CD68+ area/ total plaque area, n_1,2,3,4_=8).

**Figure S4: Infusion of omentin-1 enhanced plaque stability in Ldlr^-/-^ mice.** A. Representative images of Ldlr^-/-^ mouse’s aortic root lesion sections stained by hematoxylin and Masson's trichrome. B. Graph shows the formation of necrotic cores. Formation of necrotic cores (pointed out by dot line) was assessed by calculating the proportion of necrosis area in the plaque (n_1,2,3,4_=10). C. Graph shows the collagen content of AS plaque. Collagen content was expressed in the proportion of collagen fiber (blue) in the plaque area (n_1,2,3,4_=10). D. Graph shows the size of AS lesion in each group. No significant difference was found between experimental groups and control group (n_1,2,3,4_=10). E. Representative images of mouse’s aortic root sections stained by oil red O. F. Graph show the lipid content (red) in plaque area. Lipid content was expressed in the proportion of oil red positive area in plaque area (n_1,2,3,4_=8). All of data in this figure were presented as mean±SEM. (n.s.=non-significant, n_1_=the number of subjects in before surgery group, n_2_=the number of subjects in normal saline (NS) infusion group, n_3_=the number of subjects in group infused with omentin-1(1μg/kg/h), n_4_=the number of subjects in group infused with omentin-1(5μg/kg/h))

**Figure S5: Infusion of omentin-1 reduced inflammatory cytokines expression and cell death in plaque of Ldlr^-/-^ mouse.** A. Representative micrographs of Ldlr^-/-^ mouse’s aortic root sections stained by the antibodies against IL-1β or TNF-α. B. C. Graph show the intensity of inflammatory cytokines expression (n_1,2,3,4_=8). The results of immunohistochemistry analysis were expressed in integral optical density (IOD) of positive staining area versus plaque area ((IOD, sum)/ (Plaque area, μm^2^)). D. Aortic root samples was collected from animal models and the expression of TNF-α and IL-1β were quantified by western blot analysis. E. Representative images of mouse’s aortic root sections were stained by DAPI (blue) and the apoptotic cell was detected by TUNEL staining (green). The plaque area was marked by dot line. F. The graph shows the cells undergoing apoptosis in AS lesion. The intensity of apoptosis was quantified by counting apoptotic cells (TUNEL+ cells) in each sample (n_1,2,3,4_=8). All of data in this figure were presented as mean±SEM. (n.s.=non-significant, n_1_=the number of subjects in before surgery group, n_2_=the number of subjects in NS infusion group, n_3_=the number of subjects in group infused with omentin-1(1μg/kg/h), n_4_=the number of subjects in group infused with omentin-1(5μg/kg/h))

**Figure S6: Infusion of omentin-1 reduced macrophage infiltration in plaque of Ldlr^-/-^ mouse.** A. Representative images of mouse’s aortic root sections were stained by DAPI (blue) and the antibody of macrophage marker CD68 (red). The plaque area was marked by dot line, and the red fluorescence area within the atherosclerotic plaque represents the area of macrophage infiltration. B. The graph shows macrophage infiltration level in AS lesion. The macrophage infiltration in each sample was quantified by calculating proportion of CD68 positive area (CD68+ area/ total plaque area, n_1,2,3,4_=8). C. Mouse IgG was used as a control of the antibody against integrin receptors. Confocal analysis indicated that exogeneous omentin-1 did not exhibit co-localization with mouse IgG, which can verify the specificity of molecular interaction between omentin-1 and integrin receptors.

**Figure S7: Replicate of fluorescence analysis demonstrated the spatial co-localization of infiltrated macrophages, exogenous omentin-1 (labeled by flag tag) and integrin receptors in ApoE^-/-^ mice’s atherosclerotic plaque.** The aortic root sections were stained by primary antibodies against F4/80 (macrophage marker), αvβ3, αvβ5 and flag tag (the marker of exogenous omentin-1). Fluorescence secondary antibodies against protein from mouse, rabbit and rat were used to mark the localization of the primary antibodies. A. Macrophage (F4/80) was dyed red and αvβ3 was dyed green. The plaque area was marked by dot line. B. Macrophage (F4/80) was dyed red and αvβ5 was dyed yellow. The plaque area was marked by dot line. C. Flag-tagged exogenous omentin-1 (flag tag) was dyed red and αvβ3 was dyed green. The plaque area was marked by dot line. D. Flag-tagged exogenous omentin-1 (flag tag) was dyed red and αvβ5 was dyed yellow. The plaque area was marked by dot line. E. Flag-tagged exogenous omentin-1 (flag tag) was dyed red and macrophage (F4/80) was dyed green.

**Figure S8: Replicate of fluorescence analysis demonstrated the spatial co-localization of infiltrated macrophages, exogenous omentin-1 (labeled by flag tag) and integrin receptors in ApoE^-/-^ mice’s atherosclerotic plaque.** The aortic root sections were stained by primary antibodies against F4/80 (macrophage marker), αvβ3, αvβ5 and flag tag (the marker of exogenous omentin-1). Fluorescence secondary antibodies against protein from mouse, rabbit and rat were used to mark the localization of the primary antibodies. A. Macrophage (F4/80) was dyed red and αvβ3 was dyed green. The plaque area was marked by dot line. B. Macrophage (F4/80) was dyed red and αvβ5 was dyed yellow. The plaque area was marked by dot line. C. Flag-tagged exogenous omentin-1 (flag tag) was dyed red and αvβ3 was dyed green. The plaque area was marked by dot line. D. Flag-tagged exogenous omentin-1 (flag tag) was dyed red and αvβ5 was dyed yellow. The plaque area was marked by dot line. E. Flag-tagged exogenous omentin-1 (flag tag) was dyed red and macrophage (F4/80) was dyed green.

**Figure S9: Fluorescence analysis of co-localization of infiltrated macrophages, exogenous omentin-1 (labeled by flag tag) and integrin receptors in NS-treated ApoE^-/-^ mice’s atherosclerotic plaque.** The aortic root sections were stained by primary antibodies against F4/80 (macrophage marker), αvβ3, αvβ5 and flag tag (the marker of exogenous omentin-1). Fluorescence secondary antibodies against protein from mouse, rabbit and rat were used to mark the localization of the primary antibodies. A. Macrophage (F4/80) was dyed red and αvβ3 was dyed green. The plaque area was marked by dot line. B. Macrophage (F4/80) was dyed red and αvβ5 was dyed yellow. The plaque area was marked by dot line. C. Flag-tagged exogenous omentin-1 (flag tag) was dyed red and αvβ3 was dyed green. The plaque area was marked by dot line. D. Flag-tagged exogenous omentin-1 (flag tag) was dyed red and αvβ5 was dyed yellow. The plaque area was marked by dot line. E. Flag-tagged exogenous omentin-1 (flag tag) was dyed red and macrophage (F4/80) was dyed green. The plaque area was marked by dot line. (Lu=the lumen of aorta)

**Figure S10: Omentin-1 reduced apoptosis induced by high ox-LDL and integrin inhibitor cilengitide completely reversed its anti-apoptosis function.** RAW264.7-derived macrophages were pretreated by omentin-1 (800ng/ml) in RPMI1640 (containing 5% FBS), then they were stimulated by high ox-LDL for 24 hours. In some of the groups, cells were co-incubated with cilengitide (2.5μM) for 1 hour before adding omentin-1 to the medium. All of the cells were fixed and subjected to TUNEL analysis.

**Figure S11:** **Omentin-1 reduced high ox-LDL-induced apoptosis in RAW264.7-derived macrophages and ITGAV knockdown significantly diminished its anti-apoptosis function.** RAW264.7-derived macrophages were transfected by ITGAV siRNA or negative control (NC) for 6 hours. The medium was then replaced by RPMI 1640 (containing 5% FBS) and the cells were incubated for 12 hours. After that, cells were pretreated by omentin-1 (800ng/ml) for 1 hour and subsequently stimulated by high ox-LDL (50μg/ml) for 24 hours. All of the cells were fixed and subjected to TUNEL analysis.

**Figure S12: Omentin-1 attenuated the lipid retention induced by ox-LDL and integrin inhibitor cilengitide completely reversed its bioeffects.** RAW264.7-derived macrophages were pretreated by omentin-1 (900ng/ml) for 1.5 hours and then were co-incubated with mild ox-LDL for 24 hours. In some of the groups, cells were co-incubated with cilengitide (2.5μM) for 1 hour before adding omentin-1 to the medium. All of cells were then fixed and dyed by oil red o stain kit. The lipid droplets within the cytoplasm were dyed red.

**Figure S13: Omentin-1 attenuated the lipid retention induced by ox-LDL and integrin inhibitor cilengitide completely reversed its bioeffects.** RAW264.7-derived macrophages were pretreated by omentin-1 (900ng/ml) for 1.5 hours and then were co-incubated with mild ox-LDL for 24 hours. In some of the groups, cells were co-incubated with cilengitide (2.5μM) for 1 hour before adding omentin-1 to the medium. All of cells were then fixed and dyed by oil red o stain kit. The lipid droplets within the cytoplasm were dyed red.

**Figure S14:** A. RAW264.7-derived macrophages were transfected by ITGAV siRNA or negative control (NC) for 6 hours. The medium was then replaced by RPMI 1640 (containing 5% FBS) and the cells were incubated for 12 hours. After that, the cells were rinsed and total protein and RNAs of cells was extracted. Expression levels of ITGAV was measured by RT-qPCR analysis and western blot analysis. B. RAW264.7-derived macrophages were co-incubated with omentin-1 in different concentration for 6 hours. Some of groups were pretreated by cilengitide (2.5μM) before adding omentin-1 to the medium. Cell membrane and cytosol protein was extracted respectively for immunoblotting analysis.
