# Supplementary figures and images for: Omentin-1 Modulates Macrophage Function via Integrin Receptors αvβ3 and αvβ5 and Reverses Plaque Vulnerability in Animal Models of Atherosclerosis
Source: Front Cardiovasc Med. 2021 Nov 2;8:757926. doi: 10.3389/fcvm.2021.757926 (PMC8593239; doi:10.3389/fcvm.2021.757926)

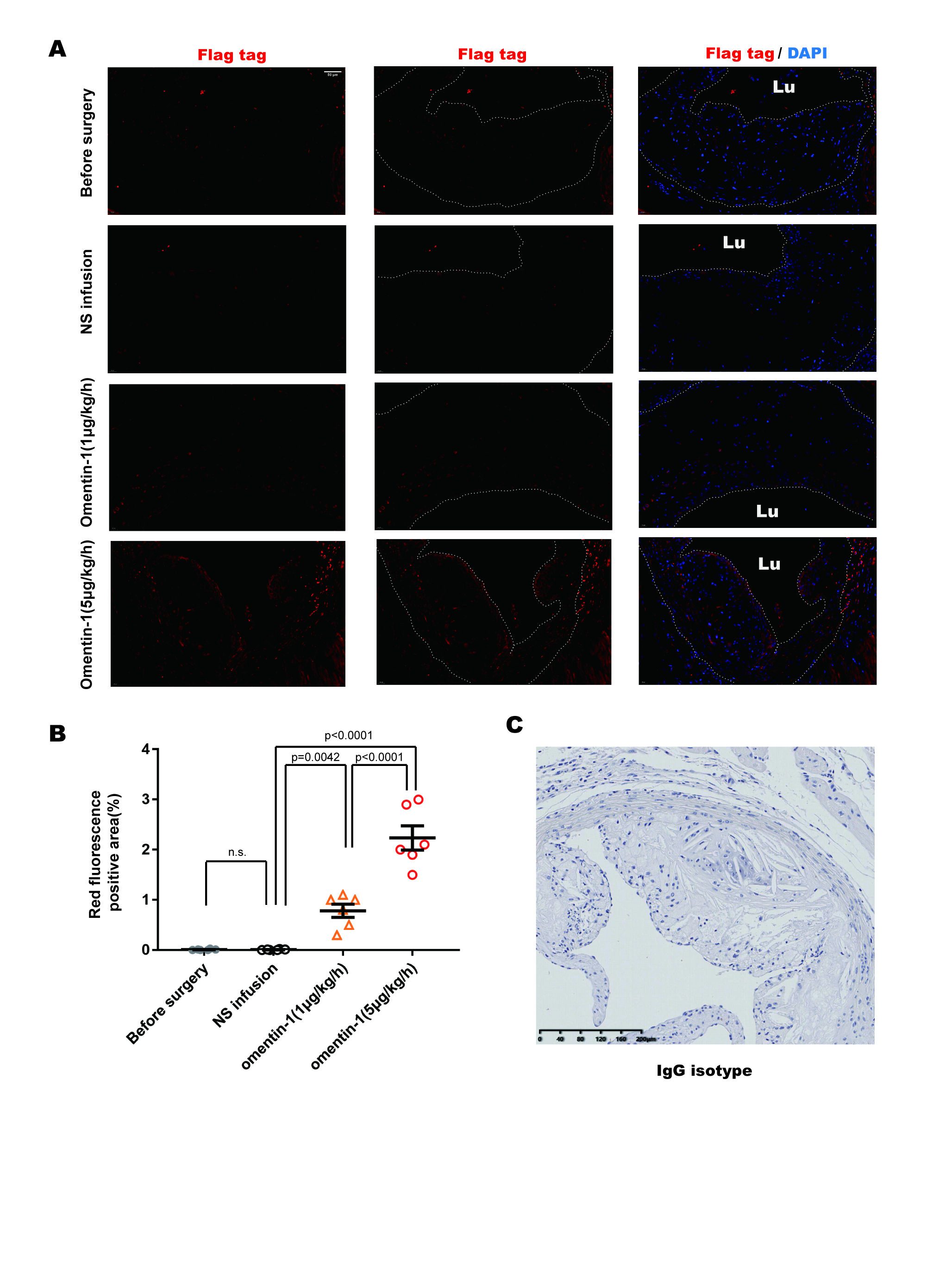

Supplement: Supplementary file 2 [file Data_Sheet_2.zip › Figure S1-S4/Fig S1.tif]

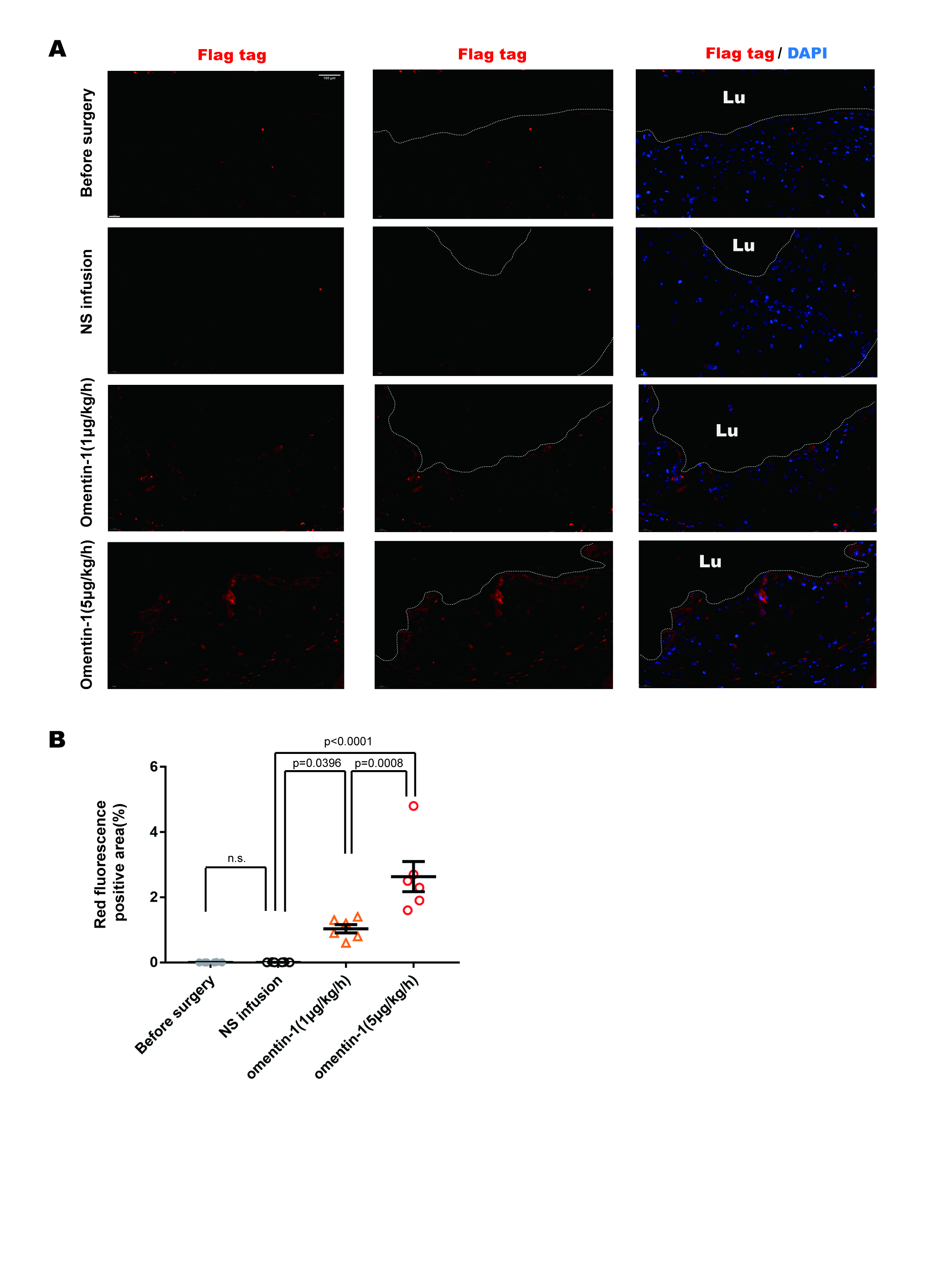

Supplement: Supplementary file 2 [file Data_Sheet_2.zip › Figure S1-S4/Fig S2.tif]

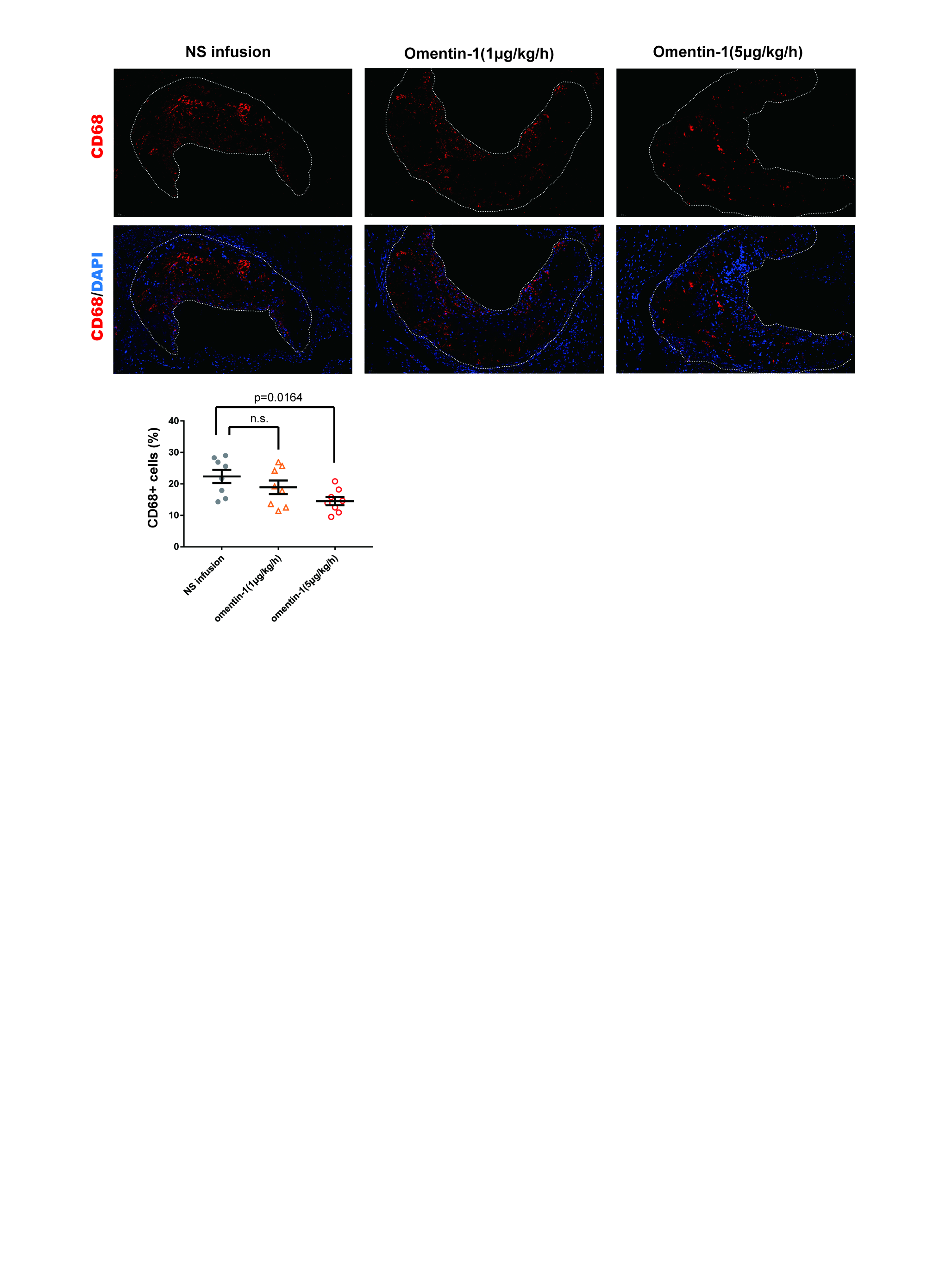

Supplement: Supplementary file 2 [file Data_Sheet_2.zip › Figure S1-S4/Fig S3.tif]

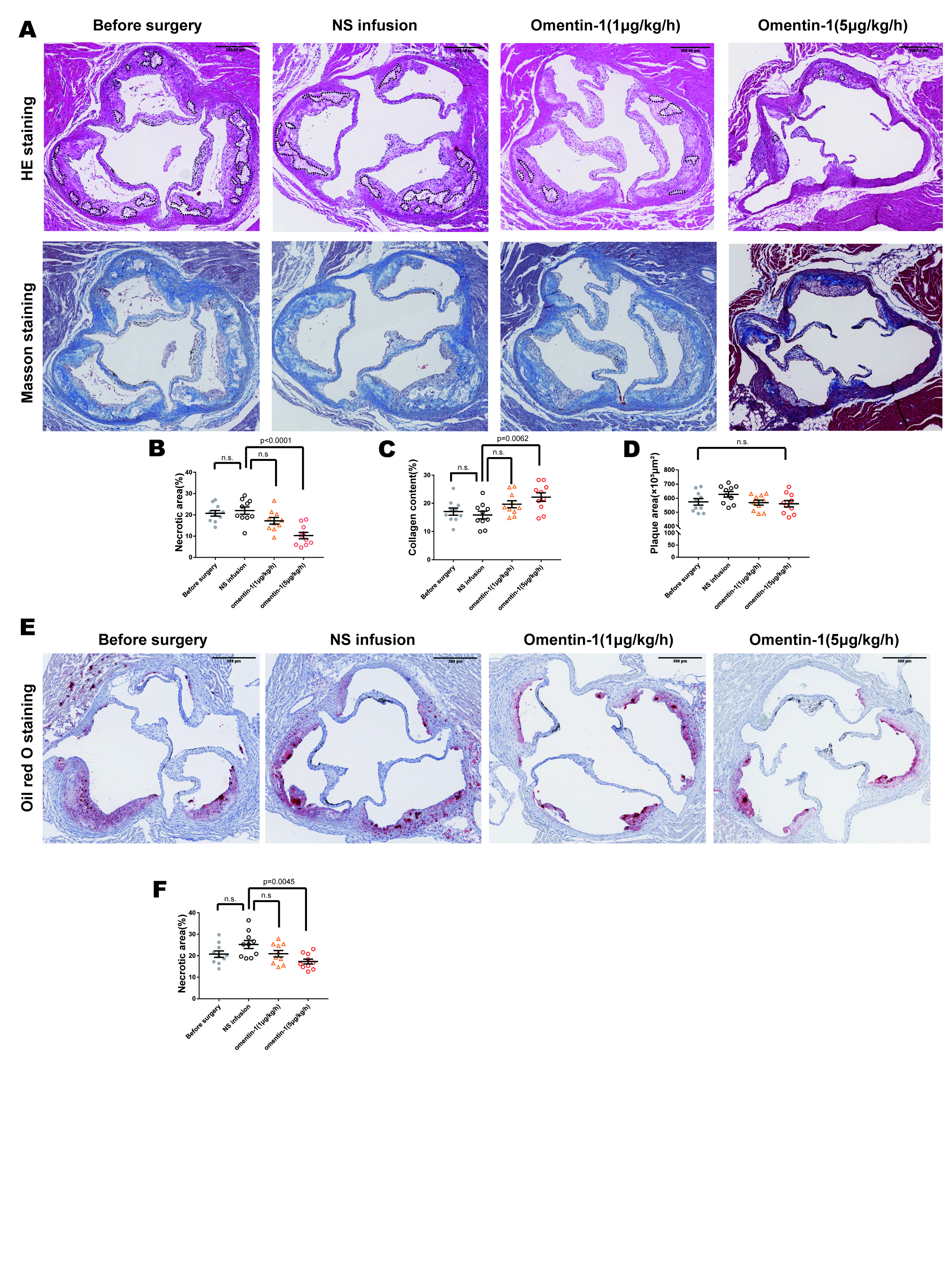

Supplement: Supplementary file 2 [file Data_Sheet_2.zip › Figure S1-S4/Fig S4.tif]

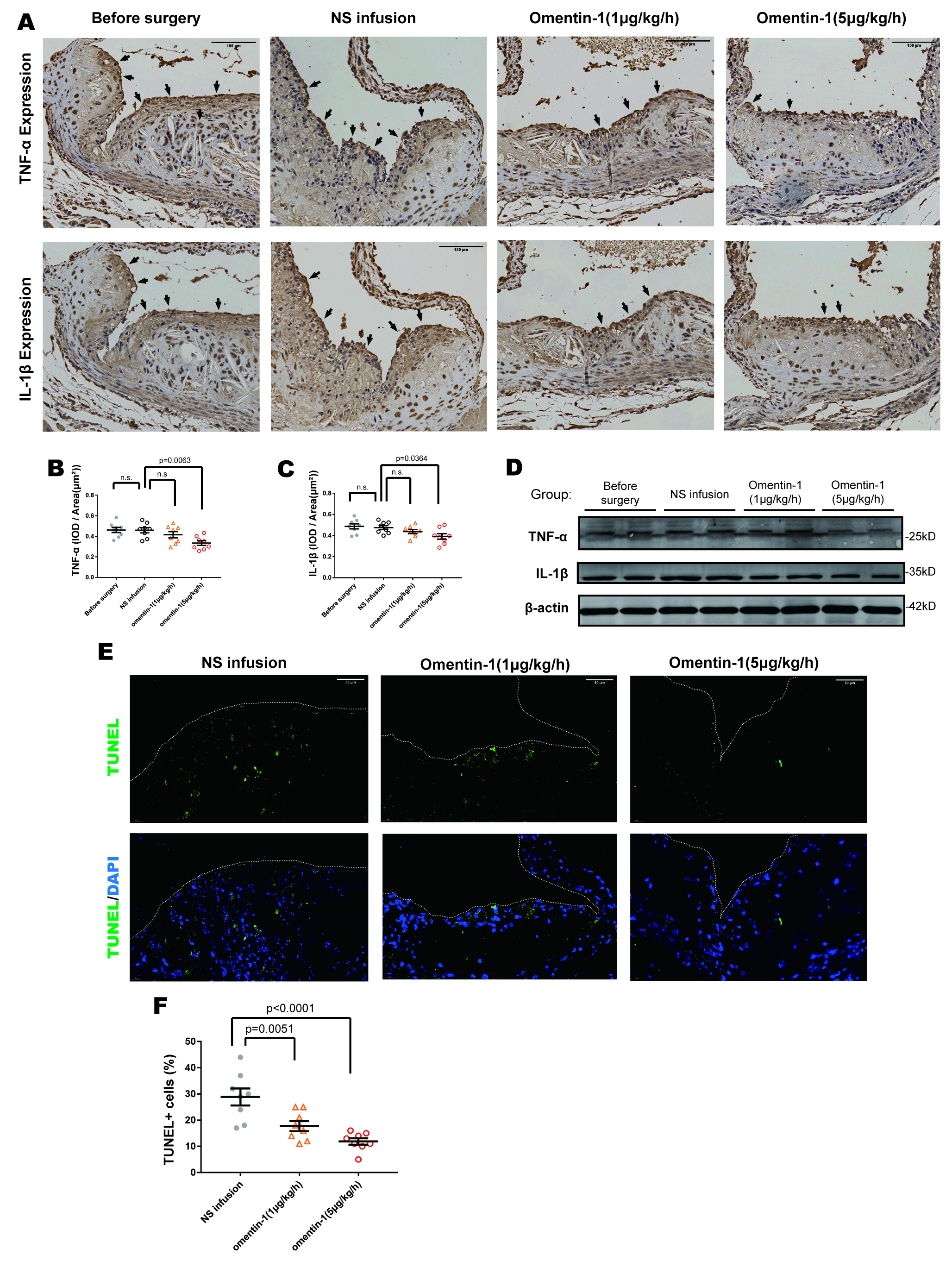

Supplement: Supplementary file 3 [file Data_Sheet_3.zip › Figure S5-S7/Fig S5.tif]

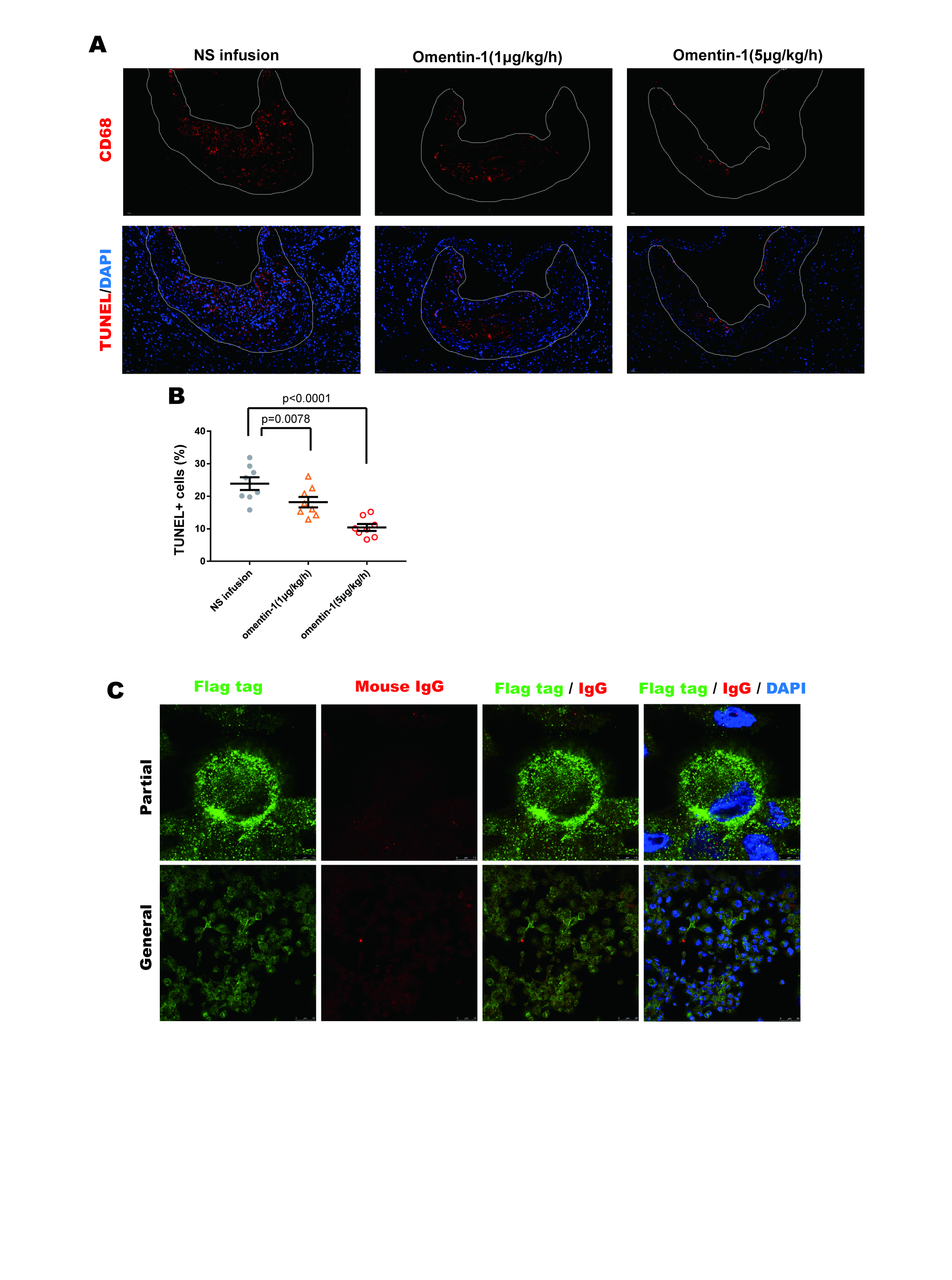

Supplement: Supplementary file 3 [file Data_Sheet_3.zip › Figure S5-S7/Fig S6.tif]

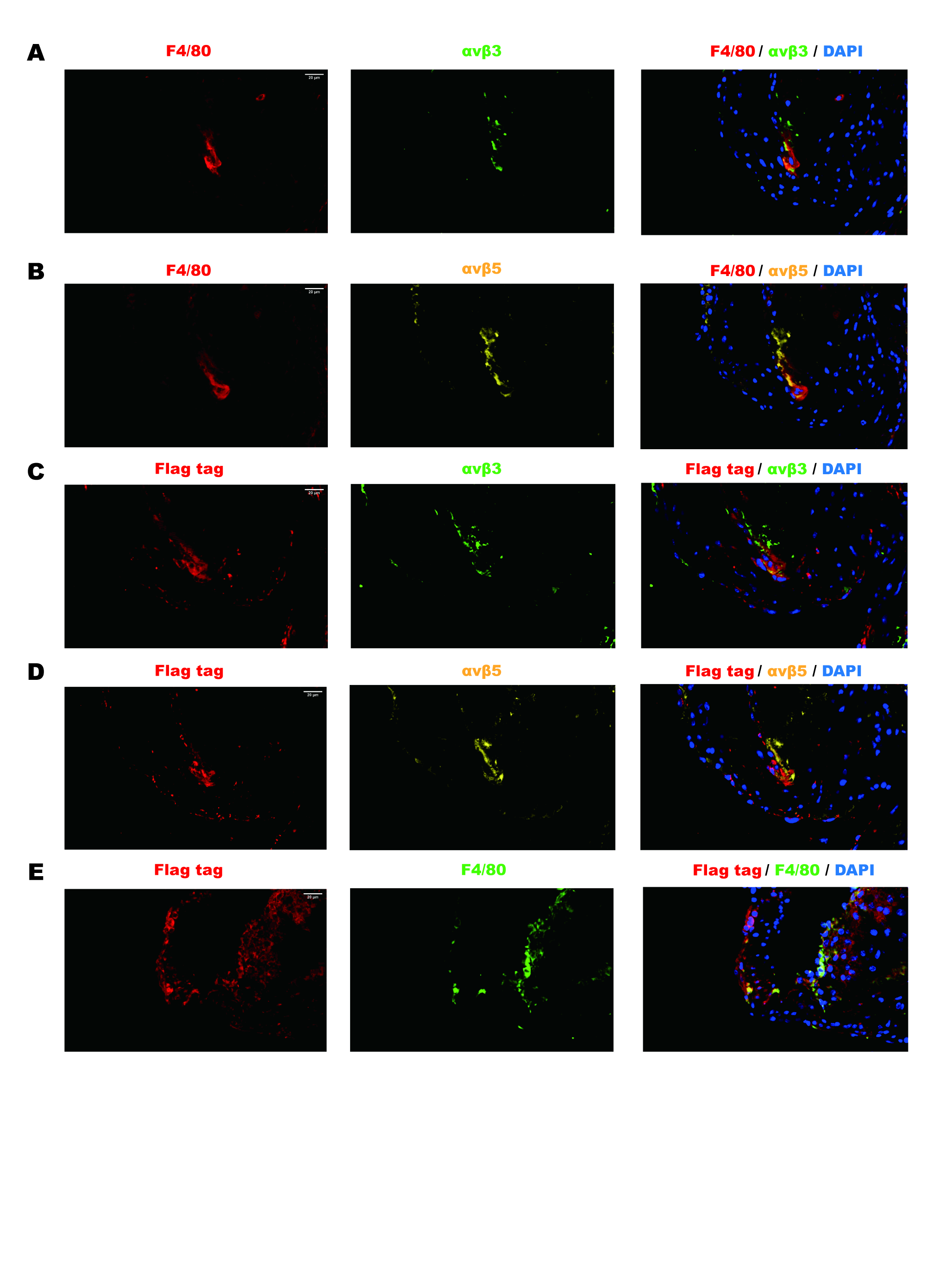

Supplement: Supplementary file 3 [file Data_Sheet_3.zip › Figure S5-S7/Fig S7.tif]

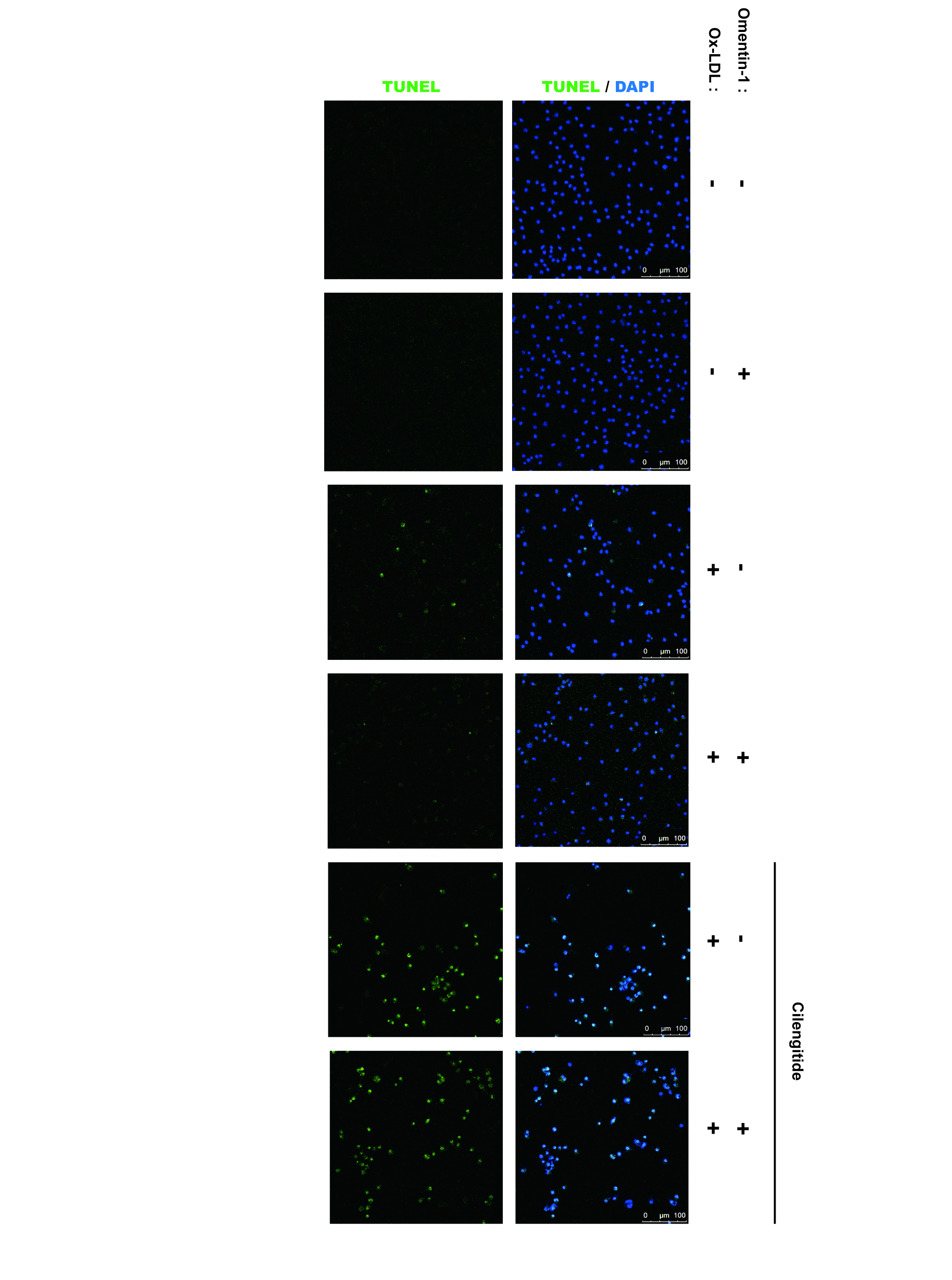

Supplement: Supplementary file 4 [file Data_Sheet_4.zip › Figure S8-S11/Fig S10.tif]

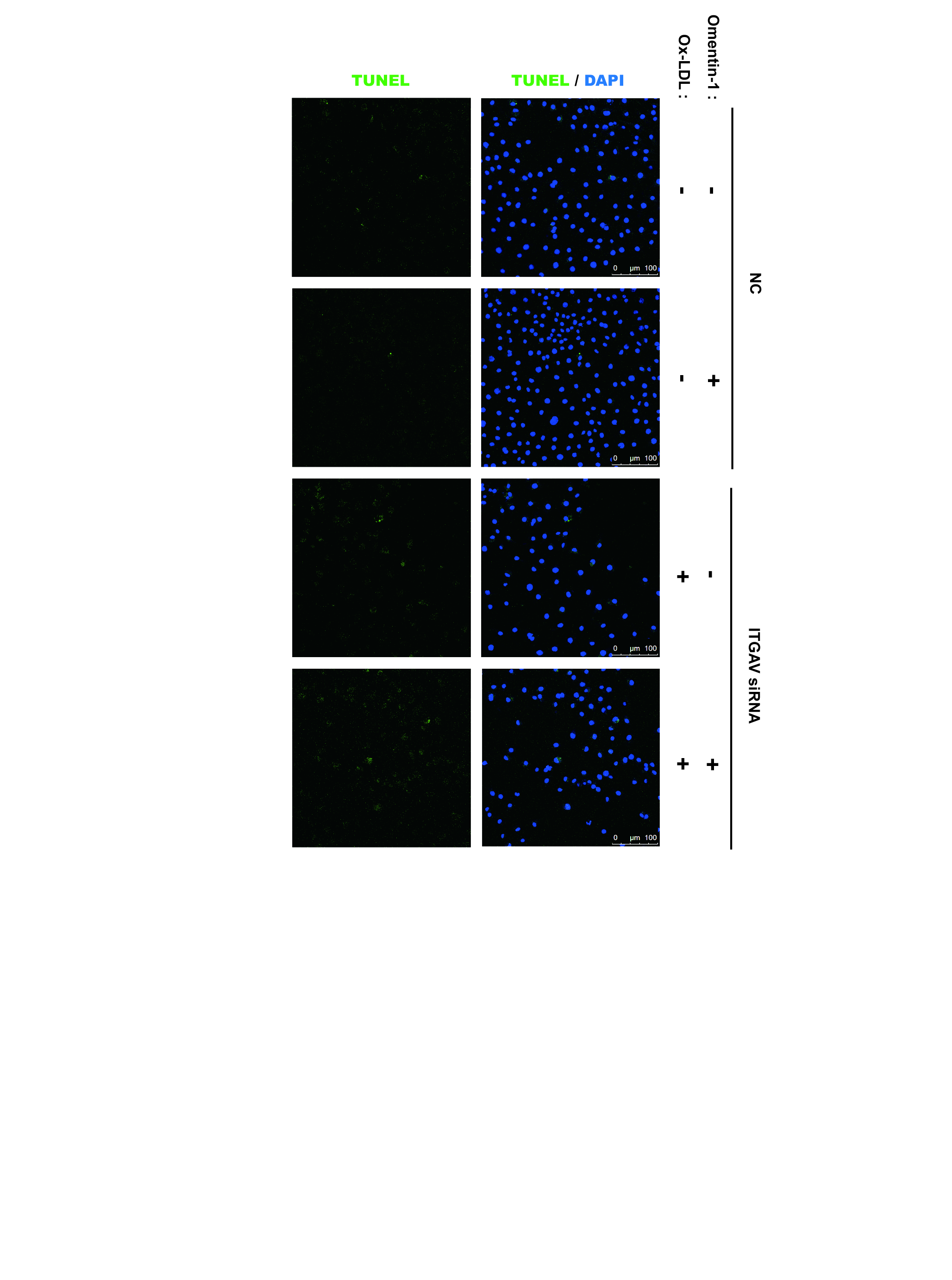

Supplement: Supplementary file 4 [file Data_Sheet_4.zip › Figure S8-S11/Fig S11.tif]

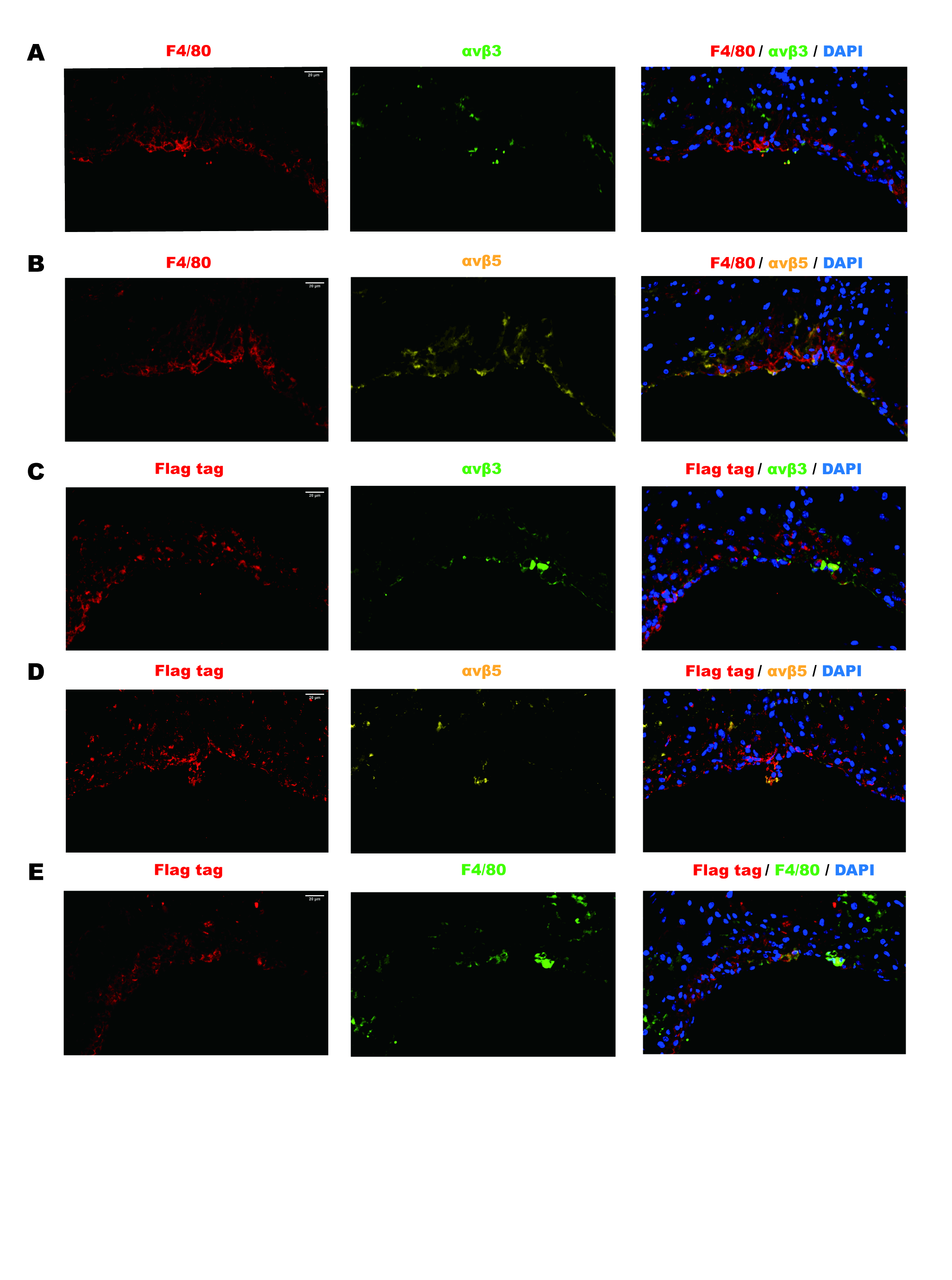

Supplement: Supplementary file 4 [file Data_Sheet_4.zip › Figure S8-S11/Fig S8.tif]

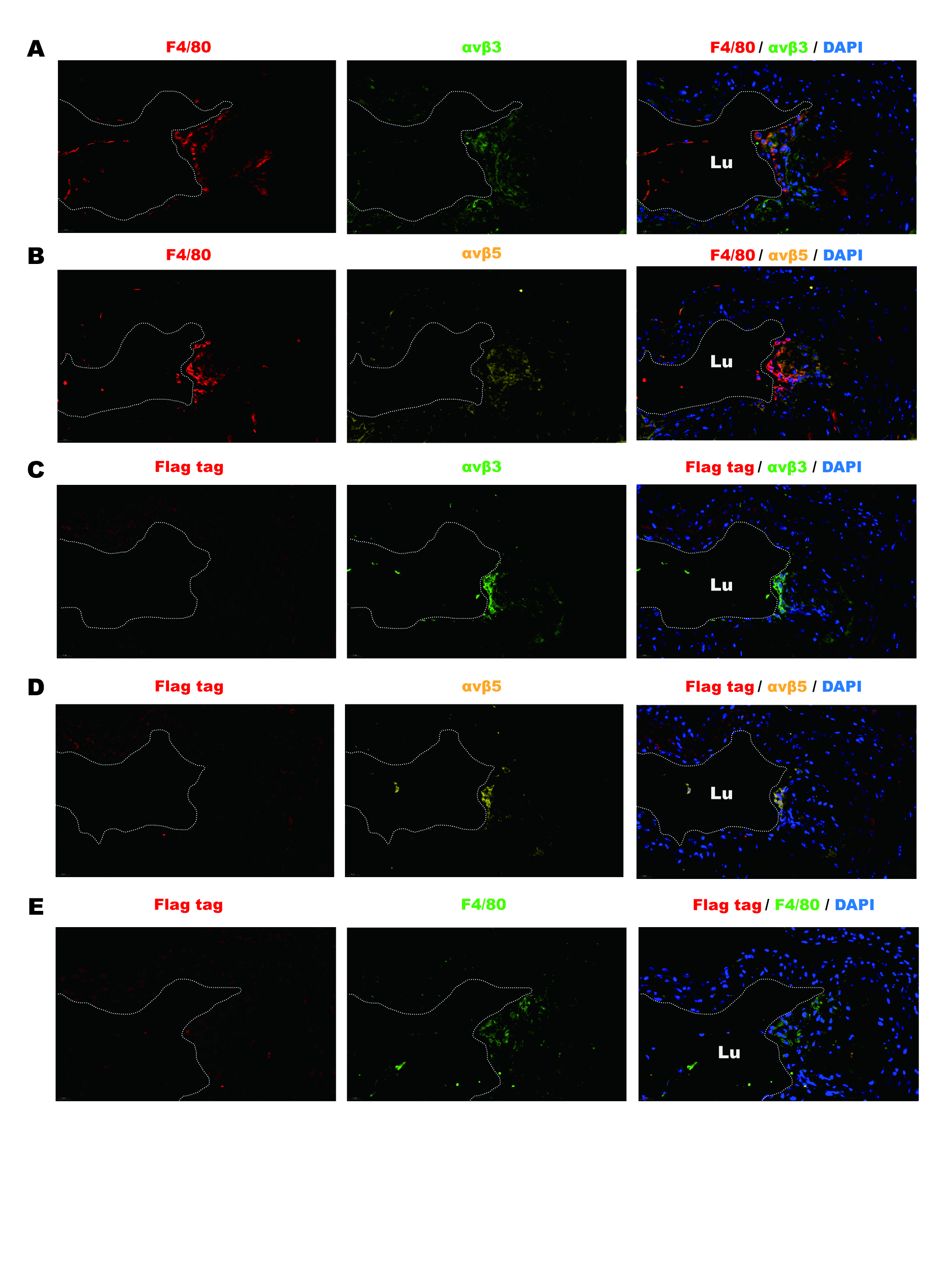

Supplement: Supplementary file 4 [file Data_Sheet_4.zip › Figure S8-S11/Fig S9.tif]

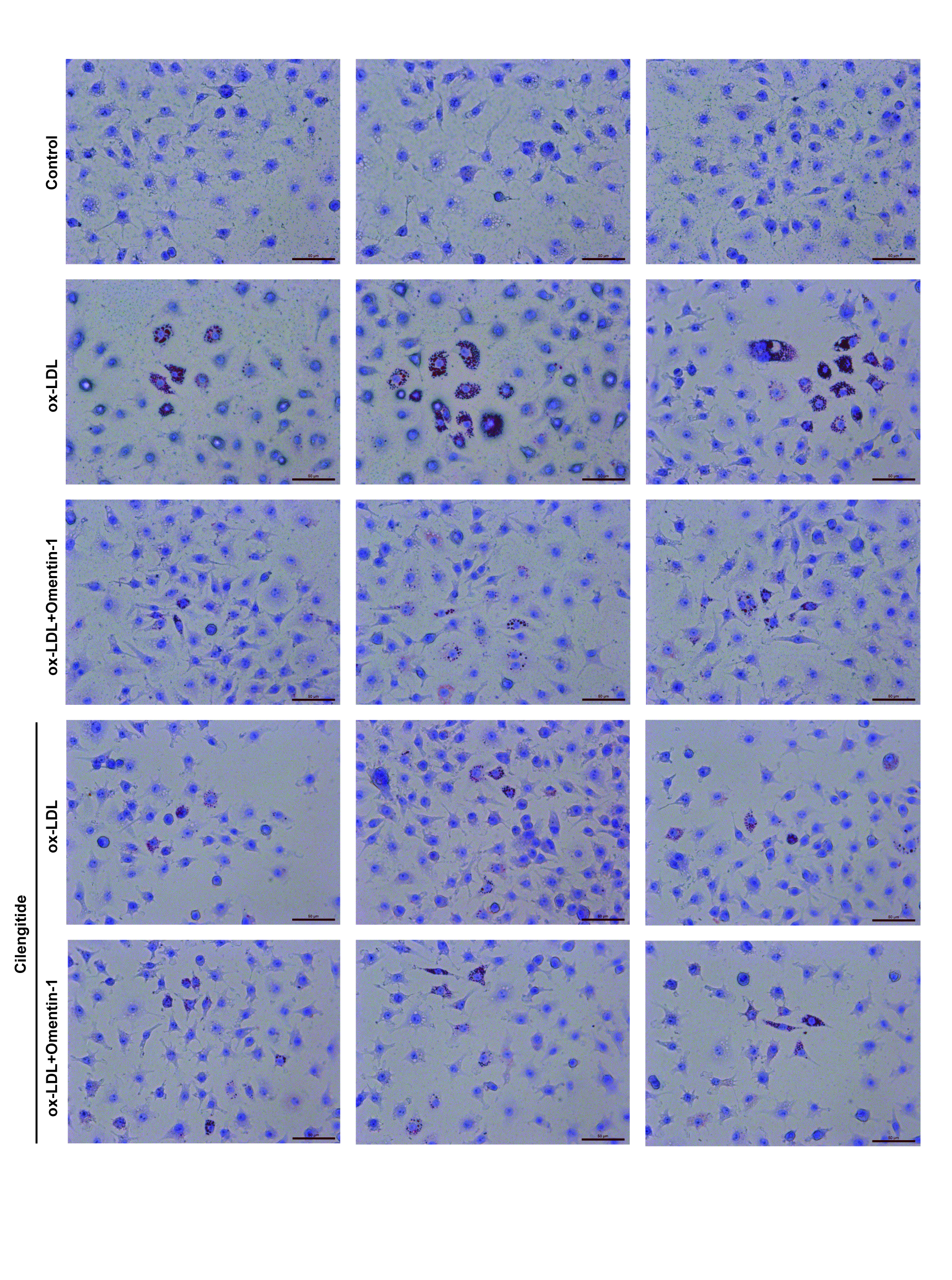

Supplement: Supplementary file 5 [file Data_Sheet_5.zip › Figure S12/Fig S12.tif]

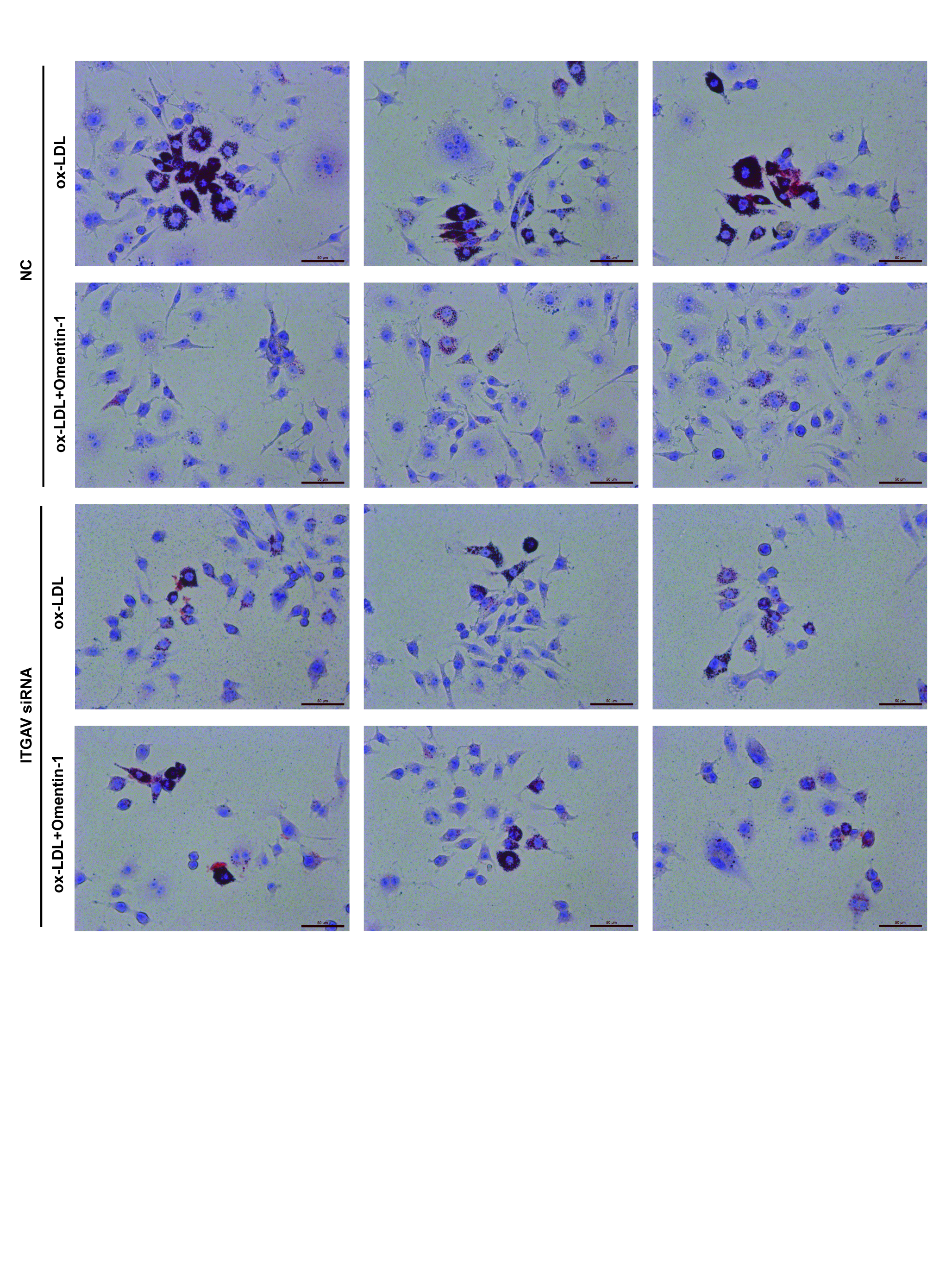

Supplement: Supplementary file 6 [file Data_Sheet_6.zip › Figure S13-14/Fig S13.tif]

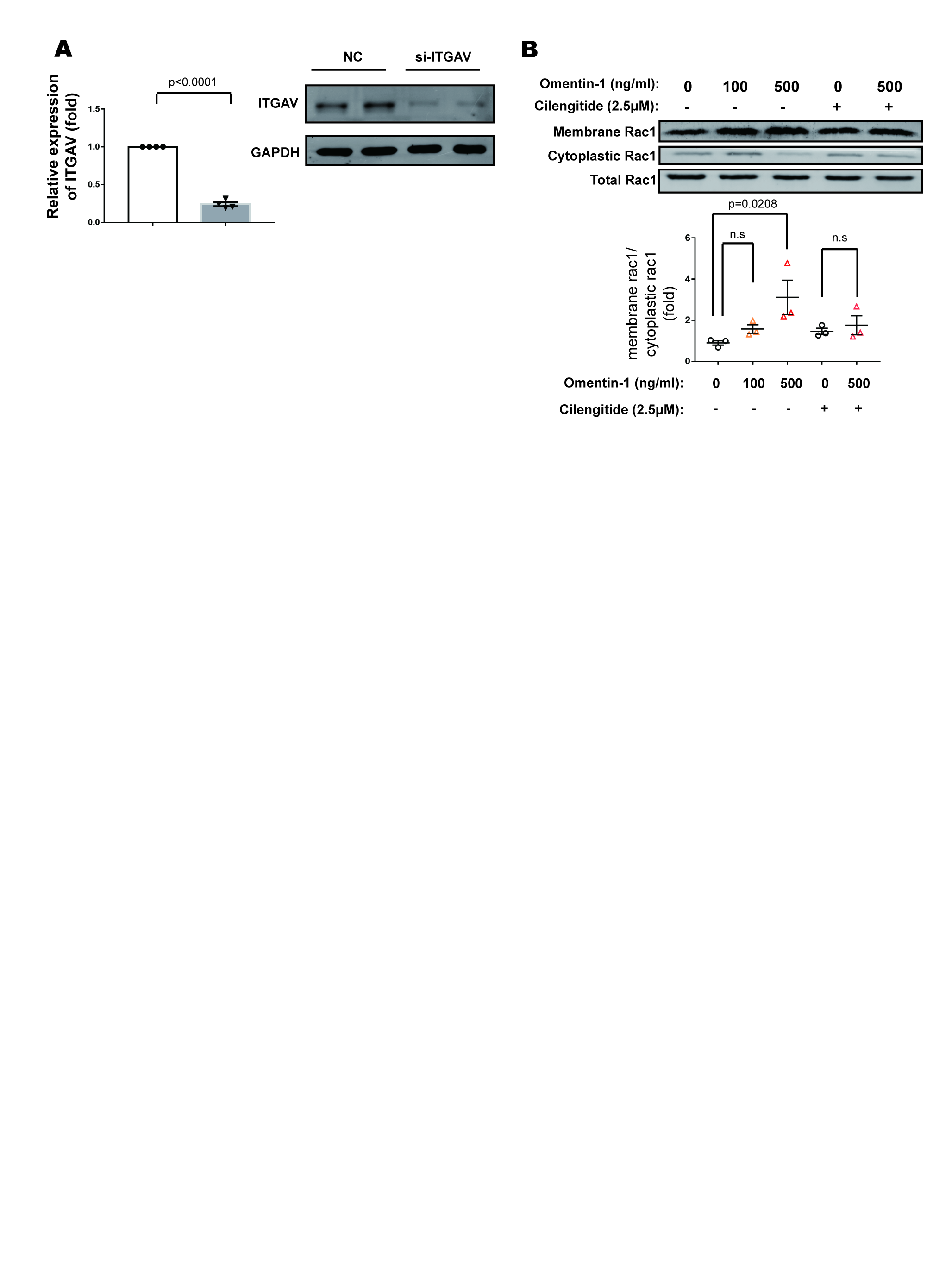

Supplement: Supplementary file 6 [file Data_Sheet_6.zip › Figure S13-14/Fig S14.tif]
