# Supplementary material for: Omentin-1 Modulates Macrophage Function via Integrin Receptors αvβ3 and αvβ5 and Reverses Plaque Vulnerability in Animal Models of Atherosclerosis
Source: Front Cardiovasc Med. 2021 Nov 2;8:757926. doi: 10.3389/fcvm.2021.757926 (PMC8593239; doi:10.3389/fcvm.2021.757926)
Supplement: Supplementary file 7 [file Data_Sheet_7.pdf]

Major Resources Table

Animals (in vivo studies)

Species	Vendor or Source	Background Strain	Sex	Persistent ID / URL
ApoE$^{-/-}$ Mouse	Beijing Vital River Laboratory Animal Technology	C57BL/6	Male	http://www.lascn.net/Item/15349.aspx
Ldlr$^{-/-}$ Mouse	Guangzhou Cyagen Biosciences Inc	C57BL/6	Male	https://www.cyagen.com/cn/zh-cn/?bd_vid=8597238793985968757

Antibodies

Target antigen	Vendor or Source	Catalog #	Working concentration	Lot # (preferred but not required)	Persistent ID / URL
αvβ3	Santa Cruz	sc-7312	IF: 1:50-1:100		https://www.scbt.com/p/integrin-alphav-beta3-antibody-23c6?requestFrom=search
αvβ5	Santa Cruz	sc-13588	IF:1:50		https://www.scbt.com/p/integrin-alphav-beta5-antibody-p1f76?requestFrom=search
αvβ3	R&D	MAB3050	Co-IP: 1:50		https://www.rndsystems.com/cn/products/human-integrin-alphavbeta3-antibody-23c6_mab3050
αvβ5	R&D	MAB2528	Co-IP:1:50		https://www.rndsystems.com/cn/products/human-integrin-alphavbeta5-antibody-p5h9_mab2528
αvβ3	Abcam	ab190147	Neutralizing: 8μg/5×10^4 cells		https://www.abcam.cn/integrin-alpha-v-beta-3-antibody-lm609-ab190147.html
αvβ5	Abcam	ab177004	Neutralizing: 8μg/5×10^4 cells		https://www.abcam.cn/integrin-alpha-vbeta-5-antibody-p1f6-ab177004.html
TNF-α	Abcam	ab9739	IHC: 1:200<br>WB: 1:1000		https://www.abcam.cn/tnf-alpha-antibody-ab9739.html
IL-1β	Abcam	ab9722	IHC: 1:400<br>WB: 1:1000		https://www.abcam.cn/il-1-beta-antibody-ab9722.html
F4/80	Abcam	ab16911	IF: 1:100		https://www.abcam.cn/f480-antibody-bm8-ab16911.html
DDDDK tag (flag tag)	Abcam	ab205606	IF: 1:100		https://www.abcam.cn/ddddk-tag-binds-to-flag-tag-sequence-antibody-epr20018-251-ab205606.html

DOI [to be added]

PE-Anti DDDDK tag (flag tag)	Abcam	ab72469	Flow cyt<br>: 1:500		https://www.abcam.cn/pe-ddddk-tag-binds-to-flag-tag-sequence-antibody-m2-ab72469.html
p-NF-kappa B p65 Ser536	Cell Signaling Technology	3033S	WB: 1:1000		https://www.cellsignal.cn/products/primary-antibodies/phospho-nf-kb-p65-ser536-93h1-rabbit-mab/3033?_=1607148869547&Ntt=phospho%20NF&tahead=true
NF-kappa B p65	Abcam	ab16502	WB: 1:1000		https://www.abcam.cn/nf-kb-p65-antibody-ab16502.html
β-actin	Cell Signaling Technology	4970S	WB: 1:1000		https://www.cellsignal.cn/products/primary-antibodies/b-actin-13e5-rabbit-mab/4970?N=4294956287&Ntt=4970s&fromPage=plp
GAPDH	Abcam	Ab9485	WB: 1:1000		https://www.abcam.cn/gapdh-antibody-loading-control-ab9485.html
Integrin αv subunit	Proteintech	27096-1-AP	WB: 1:1000		https://www.ptgcn.com/products/ITGAV-Antibody-27096-1-AP.htm
Integrin β3 subunit	Proteintech	18309-1-AP	WB: 1:1000		https://www.ptgcn.com/products/ITGB3-Antibody-18309-1-AP.htm
Integrin β5 subunit	Proteintech	28543-1-AP	WB: 1:1000		https://www.ptgcn.com/products/Integrin-beta-5-Antibody-28543-1-AP.htm
p-FAK Tyr397	Cell Signaling Technology	8556T	WB: 1:1000		https://www.cellsignal.cn/products/primary-antibodies/phospho-fak-tyr397-d20b1-rabbit-mab/8556?N=4294956287&Ntt=8556t&fromPage=plp
FAK	Abcam	ab40794	WB: 1:1000		https://www.abcam.cn/fak-antibody-ep695y-ab40794.html
p-p38 MAPK Thr180/Tyr182	Cell Signaling Technology	4511S	WB: 1:1000		https://www.cellsignal.cn/products/primary-antibodies/phospho-p38-mapk-thr180-tyr182-d3f9-xp-rabbit-mab/4511?N=4294956287&Ntt=4511s&fromPage=plp
p-38 MAPK	Cell Signaling Technology	8690S	WB: 1:1000		https://www.cellsignal.cn/products/primary-antibodies/p38-mapk-d13e1-xp-rabbitmab/8690?N=4294956287&Ntt=8690s&fromPage=plp
p-ERK Thr202/Tyr204	Cell Signaling Technology	4370S	WB: 1:1000		https://www.cellsignal.cn/products/primary-antibodies/phospho-p44-42-mapk-erk1-2-thr202-tyr204-d13-14-4e-xp-rabbit-mab/4370?N=4294956287&Ntt=4370s&fromPage=plp
ERK	Cell Signaling Technology	4695S	WB: 1:1000		https://www.cellsignal.cn/products/primary-antibodies/p44-42-mapk-erk1-2-

DOI [to be added]

					137f5rabbitmab/4695?N=4294956287&Ntt =4695s&fromPage=plp
p-Akt Thr308	Cell Signaling Technology	4056S	WB: 1:1000		https://www.cellsignal.cn/products/primary -antibodies/phospho-akt-thr308-244f9-rabbit-mab/4056?_=1618738992391&Ntt=phospho%20akt%20308&tahead=true
Akt	Cell Signaling Technology	4691S	WB: 1:1000		https://www.cellsignal.cn/products/primary-antibodies/akt-pan-11e7-rabbit-mab/4685?site-search-type=Products&N=4294956287&Ntt=akt+pan&fromPage=plp
p-AMPK Thr172	Cell Signaling Technology	50081S	WB: 1:1000		https://www.cellsignal.cn/products/primary-antibodies/phospho-ampka-thr172-d4d6d-rabbit-mab/50081?site-search-type=Products&N=4294956287&Ntt=ampk&fromPage=plp
AMPK	Cell Signaling Technology	5831S	WB: 1:1000		https://www.cellsignal.cn/products/primary-antibodies/ampka-d5a2-rabbit-mab/5831?site-search-type=Products&N=4294956287&Ntt=ampk&fromPage=plp
CD68	Cell Signaling Technology	97778S	IF: 1:200		https://www.cellsignal.cn/products/primary-antibodies/cd68-e3o7v-rabbit-mab/97778?_=1618739773380&Ntt=97778&tahead=true
Rac1	Abcam	Ab33186	WB: 1:1000		https://www.abcam.cn/rac1-antibody-23a8-ab33186.html
ITLN1(omentin-1)	Affinity Bioscience LTD.	DF12413	Co-IP: 1:100<br>WB: 1:1000		http://www.affbiotech.com/goods-15503-DF12413-ITLN1+Antibody.html

DNA/cDNA Clones

**Clone Name**	**Forward sequence (5' to 3')**	**Reverse sequence (5' to 3')**	**Source / Repository**
Mouse ITGAV primer	GTGTGAGGAACTGGTCGCCTAT	CCGTTCTCTGGTCCAACCGATA	Invitrogen
Mouse GAPDH primer	CATCACTGCCACCCAGAAGACTG	ATGCCAGTGAGCTTCCCGTTCAG	Invitrogen
ITGAV siRNA	GACCCGUUGUCACUGUAAATT	UUUACAGUGACAACGGGUCTT	GenePharma Co., Ltd (Suzhou, China)
NC (negative control) siRNA	UUCUCCGAACGUGUCACGUTT	ACGUGACACGUUCGGAGAATT	GenePharma Co., Ltd (Suzhou, China)

Cultured Cells

Name	Vendor or Source	Sex (F, M, or unknown)	Persistent ID / URL
THP1	Cyagen Bioscience (Guangzhou, China)	unknown	https://www.cyagen.com/cn/zh-cn/
RAW264.7	Cyagen Bioscience (Guangzhou, China)	unknown	https://www.cyagen.com/cn/zh-cn/

Other

Description	Source / Repository	Persistent ID / URL
Western diet	Beijing HFK Bioscience, H10141	http://www.hfkbio.com/
Mounting Medium with DAPI	Abcam, ab104139	https://www.abcam.cn/mounting-medium-with-dapi-aqueous-fluoroshield-ab104139.html
Mouse IgG1 isotype	R&D, MAB002	https://www.rndsystems.com/cn/products/mouse-igg-1-isotype-control_mab002
ALZET osmotic minipumps	ALZET Model 2004; Cupertino, CA, USA	https://www.alzet.com/
oil red o stain kit	Solarbio Life Science, G1262	http://www.solarbio.com/goods-9953.html
masson's trichrome stain kit	Solarbio Life Science, G1340	http://www.solarbio.com/goods-475.html
Cilengitide	MedChemExpress, HY-16141	https://www.medchemexpress.com/Cilengitide.html
HE staining kit	Solarbio Life Science, G1121	http://www.solarbio.com/goods-65944.html
Omentin-1 ELISA kit	Biovendor, RD191100200R	https://www.biovendor.com/omentin-1-human-elisa?d=114
Phorbol 12-myristate 13-acetate	Sigma-Aldrich, P1585	https://www.sigmaaldrich.com/catalog/product/sigma/p1585?lang=zh&region=CN
mild ox-LDL	Yiyuan Biotech, Guangzhou, YB-002	http://www.yiyuanbiotech.com/PRO.asp?id=562
high ox-LDL	Yiyuan Biotech, Guangzhou, high ox-LDL	http://www.yiyuanbiotech.com/PRO.asp?id=562
human recombinant ITLN1 (omentin-1), flag tag labeled	Abcam, ab157030<br>Lot:GR155015-11	https://www.abcam.cn/recombinant-human-itln1-protein-ab157030.html
human recombinant ITLN1 (omentin-1), his tag labeled	Abcam, ab207144	https://www.abcam.cn/recombinant-human-itln1-protein-ab207144.html

CFSE celltrace	Thermo, C34554	https://www.thermofisher.com/order/catalog/product/C34554#/C34554
Cell Tracker Deep Red Dye 630/650	Thermo, C34565	https://www.thermofisher.com/order/catalog/product/C34565#/C34565
BAY 11-7082	Abcam, ab141228	https://www.abcam.cn/bay-11-7082-ikappabalpha-kinase-inhibitor-ab141228.html
Recombinant human integrin alpha V beta 5 protein	R&D, 2528-AV-050	https://www.rndsystems.com/cn/products/recombinant-human-integrin-alpha-v-beta-5-protein-cf_2528-av
Recombinant human integrin alpha V beta 3 protein	R&D, 3050-AV-050	https://www.rndsystems.com/cn/products/recombinant-human-integrin-alpha-v-beta-3-protein-cf_3050-av
In situ cell death detection kit, fluorescence (TUNEL assay)	Roche, 11684795910	https://www.sigmaaldrich.com/catalog/product/roche/11684795910?lang=zh&region=CN
Goat anti-rabbit IgG, H&L (Alexa Fluor 488)	Abcam, ab150077	https://www.abcam.cn/goat-rabbit-igg-hl-alexa-fluor-488-ab150077.html
Goat anti-mouse IgG, H&L (Alexa Fluor 594)	Abcam, ab150116	https://www.abcam.cn/goat-mouse-igg-hl-alexa-fluor-594-ab150116.html
FITC Annexin V Apoptosis Detection Kit I	BD pharmingen, 556547	https://www.bdbiosciences.com/cn/applications/research/apoptosis/apoptosis-kits-sets/fitc-annexin-v-apoptosis-detection-kit-i/p/556547
Cell dissociation buffer, enzyme-free, PBS	Thermofisher,13151014	https://www.thermofisher.com/order/catalog/product/13151014?SID=srch-hj-13151014#/13151014?SID=srch-hj-13151014

DOI [to be added]
