# Supplementary material for: Omentin-1 Modulates Macrophage Function via Integrin Receptors αvβ3 and αvβ5 and Reverses Plaque Vulnerability in Animal Models of Atherosclerosis
Source: Front Cardiovasc Med. 2021 Nov 2;8:757926. doi: 10.3389/fcvm.2021.757926 (PMC8593239; doi:10.3389/fcvm.2021.757926)
Supplement: Supplementary file 8 [file Image_1.pdf]

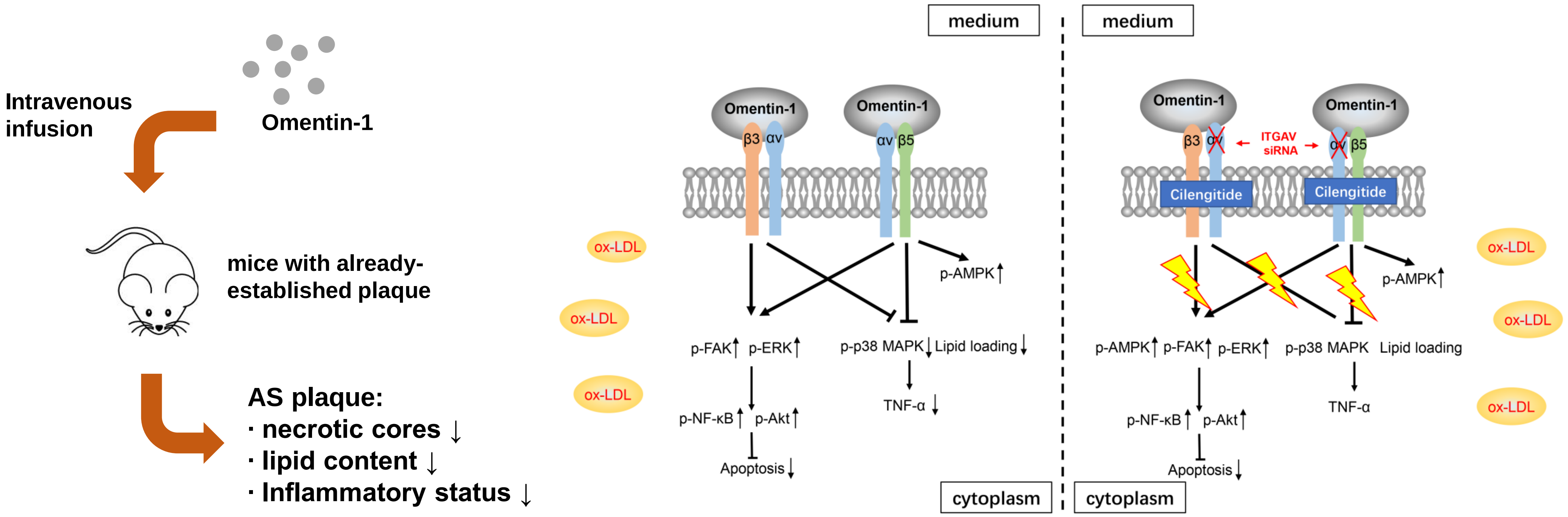

Highlights:

1.Omentin-1 infusion significantly improved the stability of already-established AS plaque in ApoE-KO mice and Ldlr-KO mice.

2.Omentin-1 attenuated apoptosis, lipid accumulation and inflammatory response induced by ox-LDL in macrophage.

3.Omentin-1 directly bound to integrin receptor αvβ3 and αvβ5 on cell membrane. Either the use of cilengitide (inhibitor of αvβ3 and αvβ5) or knocking down the expression of integrin subunit αv abrogated the bioeffects induced by omentin-1.
